# PAD as a Marker of Systemic Atherosclerosis: A Comprehensive Review of Residual Risk and Contemporary Strategies for MACE and MALE Reduction

**DOI:** 10.3390/medicina62101905

**Published:** 2026-10-02

**Authors:** Giuseppe Miceli, Maria Grazia Basso, Mariagiovanna Cuffaro, Antonino Marchese, Antonino Tuttolomondo

**Affiliations:** 1 Department of Health Promotion, Mother and Child Care, Internal Medicine and Medical Specialities (ProMISE), Università degli Studi di Palermo, Piazza delle Cliniche 2, 90127 Palermo, Italy; 2 Internal Medicine and Stroke Care Ward. University Hospital, Policlinico “P. Giaccone”, Piazza delle Cliniche 2, 90127 Palermo, Italy

**Keywords:** peripheral artery disease, MACE, MALE, chronic limb-threatening ischemia, atherosclerosis, cardiovascular risk

## Abstract

*Background*: Peripheral artery disease (PAD) is a manifestation of systemic atherosclerosis associated with substantial risks of major adverse cardiovascular events (MACE) and major adverse limb events (MALE). *Scope:* This comprehensive review examines the epidemiological and pathobiological links between PAD, polyvascular disease, and residual cardiovascular and limb risk. *Evidence Synthesis:* It summarizes contemporary evidence for integrated management through antithrombotic therapy, intensive lipid lowering, blood pressure and diabetes management, smoking cessation, structured exercise, revascularization when indicated, and longitudinal surveillance. Particular attention is given to dual-pathway inhibition, non-statin lipid-lowering agents, and cardiometabolic therapies, while distinguishing established PAD-specific benefits from subgroup or emerging evidence. *Conclusions:* A phenotype-based approach integrating ischemic, bleeding, metabolic, functional, and limb-related risk is proposed to support individualized treatment and sustained vascular protection.

## 1. Introduction

Peripheral artery disease (PAD) affects more than 236 million people worldwide and represents a major global health burden [1]. Although historically approached as a localized disorder causing claudication or limb loss, PAD is now recognized as a clinical manifestation of systemic atherosclerosis.

PAD frequently coexists with coronary and cerebrovascular disease, and its clinical course includes both major adverse cardiovascular events (MACE) and major adverse limb events (MALE). Peripheral disease severity, polyvascular involvement, diabetes, and chronic kidney disease (CKD) further amplify this risk [2].

Accordingly, revascularization for symptom relief or limb salvage must be integrated with sustained secondary prevention. Contemporary care combines appropriate antithrombotic therapy, intensive lipid lowering, cardiometabolic and blood pressure management, smoking cessation, structured exercise, and longitudinal follow-up, supported by current performance measures [3].

The present comprehensive review examines PAD as a systemic and phenotype-heterogeneous disease, evaluates residual cardiovascular and limb risk, and summarizes contemporary and emerging strategies for reducing MACE and MALE [1].

## 2. Methods

A comprehensive literature review was conducted in accordance with the Preferred Reporting Items for Systematic Reviews and Meta-Analyses (PRISMA) [4] framework to identify and select studies addressing peripheral artery disease (PAD) as a marker of systemic atherosclerosis, with particular emphasis on residual cardiovascular and limb risk, the incidence of major adverse cardiovascular events (MACE) and major adverse limb events (MALE), and contemporary strategies for risk reduction. The electronic search was performed across PubMed/MEDLINE, Embase, Scopus, and Web of Science and was supplemented by manual screening of the reference lists of relevant reviews, original articles, guidelines, consensus documents, and key source publications. Major guideline and landmark evidence sources, including ACC/AHA guideline documents, the REACH Registry, and the COMPASS PAD subanalysis, were also specifically considered.

The search strategy was based on free-text terms applied to database-specific title, abstract, keyword, or topic fields, as appropriate for each electronic database. The initial comprehensive query combined terms related to “peripheral artery disease” / “peripheral arterial disease” / “PAD” with terms addressing systemic and polyvascular atherosclerosis, cardiovascular and limb outcomes, antithrombotic therapy, lipid-lowering and cardiometabolic treatments, exercise-based interventions, prognostic stratification, and residual risk. Boolean operators (AND, OR) were used to structure the search and to retrieve literature relevant to both systemic atherosclerotic burden and cardiovascular and limb outcomes in PAD. The complete database-specific search strategies are provided in Supplementary Table S1.

The initial comprehensive search covered the period from 1 January 2000 to 20 August 2026, and yielded 10,427 records (Figure 1).

To organize the retrieved literature into clinically coherent domains and facilitate structured screening, the global strategy was subsequently subdivided into four concept-driven search domains derived directly from the primary query. These addressed: (1) PAD as a marker of systemic atherosclerosis and polyvascular disease; (2) residual risk, MACE, MALE, and prognostic stratification in PAD; (3) contemporary pharmacological strategies for cardiovascular and limb risk reduction, including antithrombotic, lipid-lowering, and cardiometabolic therapies; and (4) exercise-based and functional risk-modifying strategies, including supervised exercise therapy and rehabilitation. The terms corresponding to each of these four conceptual domains are detailed in Supplementary Table S2. This subdivision was intended to preserve the breadth of the initial search while reducing thematic overlap and improving the efficiency and consistency of the screening process.

Following removal of 3280 duplicate records, 7147 records underwent title and abstract screening. Of these, 6922 were excluded because they were clearly unrelated to the predefined scope of the review, leaving 225 reports for full-text eligibility assessment. Following full-text assessment, 88 reports were excluded because they consisted of case reports, small case series, or other publication types that did not meet the predefined eligibility criteria or did not provide evidence relevant to the clinical scope of the review or they have been considered redundant. Consequently, 137 PAD-related publications were retained for the final evidence synthesis (Figure 1). To make the search and study selection process transparent and sufficiently detailed to permit independent appraisal, clinical relevance was operationalized using explicit eligibility criteria. Publications were considered eligible when they: (1) addressed adult patients with atherosclerotic lower-extremity PAD or provided evidence directly applicable to this population; (2) examined at least one of the prespecified clinical domains of the review; (3) reported or synthesized clinically meaningful cardiovascular, limb-related, prognostic, therapeutic, or functional evidence; (4) represented an eligible source type, including randomized controlled trials, prospective or retrospective cohort studies, major registry analyses, systematic reviews, meta-analyses, international guidelines, or consensus statements; and (5) were peer-reviewed, published in English, and available in full text.

The prespecified clinical domains comprised: (1) PAD as a marker of systemic or polyvascular atherosclerosis; (2) residual cardiovascular and limb risk, including major adverse cardiovascular events, major adverse limb events, mortality, myocardial infarction, stroke, acute limb ischemia, chronic limb-threatening ischemia, major amputation, and repeat revascularization; (3) prognostic and risk-stratification approaches; and (4) contemporary pharmacological, revascularization, exercise-based, and multidisciplinary strategies aimed at improving cardiovascular, limb-related, or functional outcomes.

Case reports, small case series, editorials, commentaries, letters without original clinically relevant data, non-peer-reviewed publications, and studies focused exclusively on non-atherosclerotic peripheral vascular disorders were excluded. Studies were also excluded when they did not address any of the predefined clinical domains, did not report clinically meaningful outcomes, or provided evidence that was not directly applicable to the scope of the review.

When multiple publications addressed the same clinical question or reported overlapping evidence, priority was given to international guidelines, randomized controlled trials, systematic reviews and meta-analyses, large prospective cohorts or registries, studies with longer follow-up, and the most recent or methodologically comprehensive publication. Mechanistic studies were retained only when they provided direct biological context for clinically established associations or therapeutic strategies; mechanistic plausibility alone was not considered sufficient to support clinical recommendations.

Eligible publications comprised randomized controlled trials, prospective and retrospective cohort studies, systematic reviews, meta-analyses, international guidelines, consensus statements, and major registry-based investigations. Studies were included if they addressed one or more of the following domains: PAD, systemic or polyvascular atherosclerosis, MACE, MALE, residual cardiovascular or limb risk, prognostic stratification, or contemporary therapeutic strategies aimed at improving cardiovascular or limb outcomes. Exclusion criteria comprised case reports, very small case series, non-peer-reviewed publications, non-English-language articles, studies focused exclusively on non-atherosclerotic peripheral vascular disorders, and reports lacking clinically meaningful cardiovascular or limb outcome data. Although publications from 2000 onward were considered to ensure historical and conceptual completeness, particular emphasis was placed on evidence published from 2020 onward in order to reflect the most contemporary advances in risk stratification and treatment. The final search of all electronic databases was completed on 20 August 2026.

To enhance methodological consistency across reviewers, all investigators initially screened a common sample of 50 publications. Screening decisions were then compared, discrepancies were discussed, and the eligibility and data extraction criteria were refined before formal review initiation. Thereafter, three reviewers, working in paired and sequential fashion, independently assessed titles and abstracts, followed by full-text evaluation of potentially eligible records. Disagreements were resolved through discussion and consensus. The final set of included studies reflected broad geographic representation, although the available evidence was predominantly derived from North America and Europe, with comparatively fewer contributions from middle-income settings.

Evidence was interpreted according to a question-specific hierarchy. Therapeutic efficacy and safety conclusions were derived preferentially from international evidence-based guidelines, systematic reviews and meta-analyses of randomized trials, and individual randomized controlled trials. Prospective cohorts and large registries were primarily used to characterize epidemiology, prognosis, residual cardiovascular and limb risk, and real-world clinical practice. Retrospective and cross-sectional studies were considered supportive evidence, whereas experimental, translational, and mechanistic studies were used to provide biological context and hypothesis-generating explanations rather than to establish clinical causality or independently support therapeutic recommendations. When evidence was conflicting or heterogeneous, greater interpretative weight was assigned to studies with stronger designs, larger representative populations, clinically relevant outcomes, and longer follow-up. No formal GRADE assessment was undertaken because the article was designed as a comprehensive narrative review rather than a systematic review.

This structured approach enabled a comprehensive yet focused synthesis of the literature on PAD as a manifestation of systemic atherosclerotic disease and on contemporary strategies to mitigate its substantial residual cardiovascular and limb risk.

## 3. Pad as a Manifestation of Systemic Atherosclerotic Disease

### 3.1. Epidemiologic Burden of PAD

PAD represents a massive and escalating global cardiovascular burden. Current guideline-grade epidemiological data demonstrate that, on a global scale, the prevalence is strikingly high, with contemporary estimates ranging from 113 million to upwards of 236 million affected individuals. However, a 22% decrease in age-standardized prevalence indicates this volumetric rise is primarily driven by global population growth and demographic aging rather than an intrinsically worsening natural history. Alarmingly, while coronary artery disease (CAD) and stroke have seen a 30% reduction in age-standardized deaths, PAD-related mortality has nearly doubled over three decades, and its age-standardized mortality has remained largely unchanged [1,5,6].

The fastest growth in disease burden is occurring in low- and middle-income countries, driven by demographic transition, diabetes, tobacco exposure, and changing dietary patterns [7]. PAD also shows socioeconomic and sex-related disparities: women experience substantial functional disability, whereas men more often have premature disease-related mortality [6]. Over five years, approximately 7% of asymptomatic patients develop claudication and 21% of those with claudication progress to chronic limb-threatening ischemia (CLTI), illustrating the dynamic clinical course of PAD [2].

### 3.2. Polyvascular Disease and Systemic Risk

Patients afflicted with lower extremity arterial insufficiency carry a substantial burden of coexisting atheroma in coronary and carotid arteries, with epidemiological registries demonstrating that over half possess CAD and approximately a quarter exhibit clinically significant cerebrovascular involvement [8], underlying that PAD almost never occurs in a biological vacuum, but such a proxy for a diffuse and aggressive atherosclerotic phenotype. This multi-bed atherosclerosis state increases the longitudinal risk of MACE exponentially rather than additively [9] worsened by the concealment of silent concurrent coronary artery disease hidden by physical limitations imposed by IC preventing exertional threshold required to trigger angina [10].Even in absence of acute ischemia-related symptoms, the presence of atherosclerotic plaques in coronary, carotid or peripheral vascular three is related with highly vulnerable, pro-thrombotic systemic environment, amplifying the risk of acute ischemic events and dictating the immediate initiation of maximal, system-wide pharmacological protection. Furthermore, vulnerable plaques across the coronary, carotid, and peripheral beds induce persistent subclinical dysfunction: just as stable CAD has been appropriately reclassified as Chronic Coronary Syndrome (CCD) to reflect its dynamic, ongoing pathophysiology [11], the cerebrovascular domain is currently transitioning from the outdated label of asymptomatic carotid stenosis to the modern framework of Chronic Carotid Syndrome (CCS) [12]. For these reasons, the term “asymptomatic” may be clinically misleading in contemporary vascular practice [13]. This innovative model establishes that even without an overt history of acute myocardial infarction, ischemic stroke, or acute limb ischemia (ALI), vulnerable plaques across the coronary, carotid, and peripheral beds, through mechanisms of recurrent micro-embolization, chronic intraplaque inflammation, and downstream microvascular rarefaction, increase active atherothrombotic burden leading to end-organ damage, such as dictates progressive myocardial remodeling and ischemic heart failure, insidious vascular cognitive impairment and silent cerebral infarctions, as well as severe skeletal muscle myopathy and progressive functional decline in the lower extremities [14,15,16,17].

Atherosclerosis is a progressive inflammatory and fibroproliferative disease initiated by retention of apolipoprotein B-containing particles within the arterial wall [18]. Plaque progression, rupture, or erosion may subsequently precipitate acute thrombosis and ischemic events [19].

Because this biology affects multiple arterial territories, clinical disease in one vascular bed frequently coexists with silent or overt disease elsewhere [20]. Polyvascular disease, conventionally defined as atherosclerosis in at least two arterial territories [21], was present in one sixth of apparently single-bed cases in REACH [22], approximately 35% of asymptomatic participants in AGATHA [23], and 31% of primary-care participants in PARTNERS [24]. It has also been reported in up to 22% of patients with recent myocardial infarction [25,26]. Among patients with symptomatic PAD, additional vascular involvement exceeded 40% in EUCLID analyses [27,28] and reached 71% in tertiary vascular cohorts [29]. Each additional affected territory increases cardiovascular and limb risk [13,30].

Even apparently isolated PAD reflects systemic susceptibility. Genetic variants such as chromosome 9p21 support shared predisposition across arterial beds [31], while environmental exposures and local hemodynamics influence the first clinical presentation. Consequently, patients with PAD but no recognized coronary or cerebrovascular event remain at substantial cardiovascular risk, in some cohorts comparable to that of symptomatic coronary artery disease [32], and require comprehensive secondary prevention.

## 4. Prognostic Significance of Pad: Residual Cardiovascular and Limb Risk

### 4.1. Cardiovascular, Limb, and Mortality Risk

PAD confers a sustained risk of cardiovascular death, myocardial infarction, and ischemic stroke despite contemporary therapy [33]. Five-year cardiovascular mortality rises from approximately 9% in asymptomatic PAD to 13% in symptomatic disease, while progression to CLTI markedly increases mortality and MACE (RR, 2.26 and 1.73, respectively) [2]. Lower-extremity disease burden is also associated with systemic plaque and inflammatory burden [34], and symptomatic claudication remains prognostically important despite antiplatelet and statin therapy [27].

MALE includes acute limb ischemia (ALI), urgent revascularization, and major amputation. Its incidence is approximately 1–2% annually in unselected PAD, but rises to 5–10% over five years in symptomatic claudication and is substantially higher in CLTI [35]. Residual MALE risk persists despite standard preventive therapy [27] and is amplified by diabetes, CKD, systemic inflammation, and polyvascular disease. Major amputation is followed by three- to five-year mortality rates of 50–70% [36], and a major limb event independently predicts subsequent cardiovascular mortality [37]. In CLTI, mortality may equal or exceed that associated with several common malignancies [38]; MALE is associated with an approximately threefold risk of death and a markedly increased risk of subsequent amputation [2].

### 4.2. Polyvascular Disease and Residual Risk

Polyvascular disease, previous revascularization, and CLTI identify patients with particularly high ischemic risk [28]. Registry data show a stepwise increase in cardiovascular events with each affected arterial territory, while COMPASS and FOURIER analyses confirm higher absolute event rates and treatment benefits in patients with multiterritory disease [39,40]. These features may support treatment intensification when bleeding risk is acceptable.

Residual risk nevertheless remains multidimensional. Persistent thrombosis, inflammation, atherogenic lipoproteins, including lipoprotein(a) and triglyceride-rich remnants, and inadequately controlled risk factors may continue to drive events despite conventional antiplatelet and lipid-lowering therapy [27,41]. Treatment should therefore address the dominant modifiable pathways without inferring clinical benefit from mechanistic plausibility alone.

## 5. Pathobiological Basis of Residual Risk in Pad

### 5.1. Atherothrombosis and Platelet-Coagulation Activation

The pathophysiological mechanisms underlying residual risk in PAD extend beyond the conventional paradigm of progressive luminal narrowing. Contemporary epidemiological evidence suggests that traditional modifiable risk factors account for approximately 55% of the global disease burden, indicating that additional biological and clinical determinants may contribute substantially to disease progression and adverse outcomes [21]. The lower-extremity arterial circulation is exposed to complex hemodynamic shear stress and biomechanical forces, particularly at arterial bifurcations, which may promote endothelial dysfunction and facilitate the interaction of circulating platelets and coagulation factors with thrombogenic components of the vascular wall. These processes can contribute to sustained platelet activation and thrombin generation and may partly explain why clinically relevant residual cardiovascular and limb risk persists despite standard single-antiplatelet therapy [42]. In addition to its role in thrombus formation, thrombin may influence vascular remodeling through the activation of protease-activated receptors, thereby promoting smooth muscle cell proliferation, extracellular matrix deposition, and intimal hyperplasia. The interplay between platelet activation, coagulation, inflammation, and vascular remodeling may therefore contribute to both acute atherothrombotic events and longer-term disease progression, including after technically successful revascularization. This pathophysiological framework provides a plausible rationale for therapeutic strategies targeting complementary components of the thrombotic pathway. Accordingly, in appropriately selected patients, dual-pathway inhibition may reduce residual cardiovascular and limb risk by attenuating both platelet-mediated and coagulation-mediated mechanisms, although its use should be guided primarily by evidence from randomized clinical trials and by individualized assessment of ischemic and bleeding risks [30].

### 5.2. Lipid-Driven Residual Risk

Residual lipid risk reflects the cumulative arterial exposure to apolipoprotein B-containing particles and may persist despite substantial reductions in LDL-C. LDL-C quantifies the cholesterol content carried predominantly within LDL particles but does not directly represent the total number of circulating atherogenic particles. Non-HDL-C and apolipoprotein B may therefore provide complementary information, particularly in patients with diabetes, hypertriglyceridaemia, obesity, or metabolic dysfunction, in whom cholesterol-rich remnant particles may contribute to risk that is not fully captured by LDL-C alone [43].

Lipoprotein(a) represents an additional, predominantly genetically determined component of residual risk through atherogenic, inflammatory, and potentially prothrombotic pathways [44]. In patients with PAD, elevated lipoprotein(a) has been associated with increases of up to 30% in MACE risk and 19% in MALE risk [43,45]. Mendelian-randomization data additionally implicate small, dense very-low-density lipoprotein particles in the development of polyvascular disease, supporting the broader relevance of triglyceride-rich lipoproteins and remnant cholesterol [46]. These observations support a more comprehensive characterization of atherogenic lipid burden in selected high-risk patients, although the extent to which targeting individual lipid fractions translates into PAD-specific clinical benefit varies according to the intervention and available evidence.

### 5.3. Inflammation and Immune Dysregulation

Atherosclerosis is sustained by interactions between lipid accumulation, endothelial dysfunction, innate and adaptive immune activation, and thrombosis. Inflammatory pathways may contribute to plaque progression, impaired endothelial repair, and vulnerability to clinical events; however, the relative contribution of these mechanisms varies among patients and across different stages of PAD. Elevated high-sensitivity C-reactive protein and interleukin-6 have been associated with higher-risk vascular phenotypes [47], while increasing biomarker concentrations in multiterritory disease suggest a relationship between systemic inflammatory activity and overall atherosclerotic burden [48].

These biomarkers provide evidence of ongoing immune activation and may offer prognostic information beyond conventional risk factors [49]. Nevertheless, they are nonspecific, can be influenced by infection, CKD, obesity, smoking, and other comorbidities, and are not currently sufficient to determine treatment selection in routine PAD practice. Although anti-inflammatory pathways represent a biologically plausible therapeutic target, the clinical utility of inflammatory biomarkers for directing PAD-specific treatment and the balance between vascular benefit and adverse effects require further evaluation in dedicated studies [50].

### 5.4. Insulin Resistance, Diabetes, and Metabolic Dysfunction

Diabetes and insulin resistance accelerate the development and progression of PAD through multiple interacting pathways, including endothelial dysfunction, advanced glycation, oxidative stress, chronic low-grade inflammation, impaired fibrinolysis, and adverse changes in lipoprotein composition. Diabetes is also associated with microvascular dysfunction and more diffuse, distal arterial involvement, which may limit collateral perfusion and complicate revascularization. These abnormalities contribute to increased cardiovascular risk while simultaneously impairing tissue oxygenation, wound healing, and resistance to infection.

The clinical expression of PAD may be altered by peripheral sensory neuropathy, which can attenuate claudication and ischemic pain despite progressive tissue hypoperfusion. Consequently, diabetic patients may present with atypical symptoms or be identified only after the development of ulceration, infection, or more advanced limb disease. Functional assessment, pulse examination, foot inspection, and appropriate physiological testing are therefore particularly important in this population.

Coexisting CKD further amplifies cardiovascular and limb risk through anaemia, inflammation, endothelial dysfunction, disturbances in mineral metabolism, and arterial calcification. It may also affect the reliability of conventional diagnostic measurements and restrict therapeutic options through increased procedural, renal, and bleeding risks [51]. Diabetes and CKD should therefore be considered interacting risk amplifiers rather than isolated comorbidities, requiring coordinated metabolic, renal, cardiovascular, and limb-directed management.

### 5.5. CKD as a Risk Amplifier

In parallel with metabolic dysfunction, CKD represents an important risk amplifier and is strongly associated with medial arterial calcification, a pathological process commonly referred to as Mönckeberg sclerosis. Unlike the lipid-rich intimal plaques characteristic of atherosclerosis, medial calcification involves osteogenic transdifferentiation of vascular smooth muscle cells and is promoted by multiple abnormalities associated with the uremic milieu, including disturbances in calcium–phosphate homeostasis and elevated circulating levels of fibroblast growth factor 23 [52]. This process can increase arterial stiffness and reduce the compliance of lower-extremity arteries, particularly in patients with advanced CKD [53]. A substantial medial calcific burden may impair distal hemodynamics and increase the technical complexity of endovascular interventions by limiting balloon expansion, stent deployment, and vessel recoil control. Severe calcification has consequently been associated with lower procedural success, reduced intervention durability, restenosis, and an increased risk of major adverse limb outcomes, including amputation; however, these outcomes remain heterogeneous and are also influenced by lesion characteristics, disease severity, comorbidities, and the revascularization strategy employed. To address this anatomical challenge, contemporary vascular practice increasingly incorporates advanced vessel-preparation techniques, including intravascular lithotripsy. By delivering acoustic pressure waves intended to modify vascular calcium and improve vessel compliance before adjunctive treatment, intravascular lithotripsy may facilitate endovascular revascularization in selected patients with heavily calcified lesions, although its effects on long-term clinical outcomes require continued evaluation [54].

In patients with diabetes or CKD, the typical symptomatic manifestations of PAD may be attenuated or absent. Peripheral sensory neuropathy can reduce the perception of ischemic pain, thereby limiting the clinical sensitivity of intermittent claudication as an early warning symptom. Consequently, PAD may remain unrecognized until more advanced stages, and some patients first present with CLTI, tissue loss, or gangrene. Nevertheless, the timing and severity of presentation vary considerably according to the extent of vascular disease, the presence of neuropathy, functional status, access to care, and the intensity of cardiovascular surveillance.

Diagnostic assessment may be further complicated by medial arterial calcification, which can render distal arteries incompressible and produce falsely elevated ankle pressures, thereby reducing the reliability of the resting ankle–brachial index (ABI). Accordingly, contemporary guidelines recommend a stepwise diagnostic approach tailored to the clinical context. When the ABI is non-compressible or exceeds 1.40, measurement of the toe–brachial index (TBI), with or without additional physiological or imaging modalities, is recommended to assess distal perfusion more accurately. In patients with exertional lower-extremity symptoms and a normal or borderline resting ABI, exercise ABI testing should be performed to investigate otherwise occult hemodynamic impairment [1].

### 5.6. Microvascular Dysfunction and Tissue-Level Ischemic Vulnerability

Microvascular dysfunction may limit tissue oxygenation and wound healing even after macrovascular patency has been restored [38]. Capillary rarefaction, endothelial dysfunction, impaired angiogenesis, and cellular abnormalities contribute to this mismatch [55]. Tissue-level assessment with toe pressures, transcutaneous oxygen measurements, or selected perfusion imaging may complement anatomical imaging in CLTI [56].

### 5.7. PAD as a Phenotype-Heterogeneous Syndrome

PAD is therefore a heterogeneous syndrome in which atherothrombotic, lipid, inflammatory, metabolic, calcific, and microvascular mechanisms vary among patients [52,57]. These mechanisms can inform risk interpretation, but treatment selection should remain anchored to clinical phenotype and outcome evidence; biomarker-defined or mechanistic subtypes remain investigational [51,58].

## 6. Clinical Phenotypes and Risk Stratification in Pad

For clinical decision-making, PAD can be considered across asymptomatic disease, chronic symptomatic PAD, CLTI, ALI, and the post-revascularization state. These phenotypes differ in immediate objectives, cardiovascular and limb risk, and the intensity of surveillance required [1].

### 6.1. Asymptomatic PAD

Apparently asymptomatic PAD should not be interpreted as biologically inactive or clinically irrelevant disease. The absence of typical exertional symptoms may reflect reduced habitual activity, diabetic or age-related neuropathy, musculoskeletal limitations, heart failure, pulmonary disease, or an inability to walk sufficiently to provoke claudication. Careful clinical assessment should therefore explore walking capacity, atypical exertional symptoms, pulse abnormalities, foot integrity, and potential barriers that may conceal functional impairment.

A reduced resting ankle–brachial index (ABI) identifies lower-extremity atherosclerosis and is associated with increased cardiovascular risk even in the absence of classical claudication. Its detection should prompt comprehensive management of smoking, atherogenic lipid levels, BP, diabetes, physical inactivity, and other modifiable risk factors [1]. Antithrombotic therapy should be considered according to the overall cardiovascular and bleeding profile rather than prescribed solely on the basis of an abnormal ABI. Exercise and walking should be encouraged when feasible, whereas revascularization is not indicated solely to prevent progression in an otherwise asymptomatic limb. Periodic reassessment is appropriate because declining mobility, neuropathy, or progressive disease may alter the apparent clinical phenotype over time.

### 6.2. Chronic Symptomatic PAD and Claudication

Chronic symptomatic PAD encompasses a spectrum ranging from atypical exertional leg symptoms to classical intermittent claudication. Although limb viability is generally preserved, walking impairment may substantially restrict independence, occupational activity, and quality of life. Functional limitation results not only from reduced conduit-artery blood flow but also from endothelial dysfunction, impaired muscle perfusion, mitochondrial abnormalities, skeletal-muscle deconditioning, and altered gait. Symptom severity may therefore correlate imperfectly with the anatomical extent of arterial obstruction.

Management should pursue cardiovascular prevention and functional recovery in parallel. Guideline-directed preventive therapy should be combined with structured exercise, preferably supervised exercise therapy when available, together with smoking cessation and management of relevant comorbidities. Clinical response should be assessed using walking performance, daily activity, and patient-reported functional limitation rather than haemodynamic measurements alone [42]. Revascularization may be considered when symptoms remain lifestyle-limiting despite appropriate medical therapy and structured exercise, provided that the expected functional benefit outweighs procedural risk. The decision should incorporate lesion anatomy, comorbidity, baseline mobility, patient preferences, and the likelihood that nonvascular conditions contribute to the reported limitation.

### 6.3. CLTI

CLTI represents the most advanced clinical manifestation of PAD. It is characterized by ischemic rest pain, non-healing ulceration, or gangrene attributable to objectively documented arterial occlusive disease and is associated with a poor short-term prognosis, including high rates of major amputation, cardiovascular events, and mortality [59]. Rather than resulting solely from an isolated arterial occlusion, CLTI commonly reflects the interaction of extensive macrovascular disease, impaired microcirculatory perfusion, and multiple risk-amplifying conditions. Long-standing diabetes mellitus, advanced CKD or end-stage kidney disease, and extensive infrapopliteal medial calcification are particularly prevalent in this population and may contribute to both disease severity and therapeutic complexity [38].

In highly vulnerable patients, impaired tissue perfusion may interact with sensory neuropathy, reduced local immune defenses, and impaired wound healing, allowing minor foot trauma to progress to complex neuroischemic lesions. CLTI should therefore be considered not only as a localized manifestation of lower-extremity arterial disease but also as a marker of advanced systemic atherosclerotic burden. Patients with CLTI frequently have concomitant coronary and cerebrovascular disease, which contributes substantially to their elevated risk of myocardial infarction, stroke, and cardiovascular death [60]. In addition, necrotic tissue and chronic wound infection may promote systemic inflammatory and prothrombotic responses through the release of acute-phase mediators, reactive oxygen species, and inflammatory cytokines. These mechanisms could potentially contribute to the heightened cardiovascular risk observed in CLTI; however, the relationship is likely multifactorial, and the available evidence does not establish that wound-related inflammation directly destabilizes distant coronary or carotid plaques or independently causes subsequent ischemic events [61].

Given its substantial limb-related and systemic risk, CLTI requires timely, multidisciplinary assessment and individualized management. Clinical decision-making should integrate patient-level procedural risk, the severity of limb threat, and the anatomical pattern of arterial disease. The Society for Vascular Surgery Wound, Ischemia, and foot Infection (WIfI) classification provides a structured assessment of limb-threat severity and estimated amputation risk by grading wound extent, ischemia, and foot infection. The Global Limb Anatomic Staging System (GLASS) complements this clinical assessment by characterizing the anatomical complexity of infrainguinal disease along a selected target arterial path and by estimating the expected technical durability of revascularization. WIfI and GLASS therefore provide complementary clinical and anatomical information that can support, but not replace, multidisciplinary judgment when selecting the most appropriate revascularization strategy [38].

Revascularization should be considered when technically feasible and clinically appropriate, with the choice between surgical and endovascular approaches informed by the patient’s overall risk, WIfI stage, GLASS-defined anatomy, conduit availability, anticipated procedural durability, functional status, and treatment goals. Revascularization should be integrated with optimal wound care, infection control, cardiovascular risk-factor modification, and evidence-based pharmacological therapy. This coordinated approach aims to preserve limb viability, promote wound healing, reduce major adverse cardiovascular and limb events, and improve overall survival.

### 6.4. Post-Revascularization PAD

Lower-extremity revascularization modifies limb perfusion but does not eliminate the underlying atherosclerotic and thrombotic risk. Patients remain vulnerable to restenosis, graft or device failure, acute limb ischemia (ALI), recurrent symptoms, and cardiovascular events, with the early post-procedural period representing a particularly relevant phase for limb-related complications. Follow-up should therefore include reassessment of symptoms, wound healing, pulse examination, functional recovery, and adherence to preventive therapy. ABI measurement and duplex ultrasound should be performed when clinically indicated and incorporated into structured surveillance protocols after procedures in which anatomical patency may influence subsequent management [62].

Post-revascularization care should also include periodic reconsideration of the antithrombotic regimen according to procedural characteristics, time elapsed since intervention, concomitant coronary indications, renal function, and bleeding risk. Lipid-lowering therapy, smoking cessation, BP and glycemic control, exercise or rehabilitation, and preventive foot care should be maintained irrespective of initial technical success. Contemporary quality measures consequently emphasize longitudinal clinical and vascular surveillance rather than equating restoration of arterial patency with resolution of the disease process [3,63].

### 6.5. Polyvascular PAD

Polyvascular PAD reflects a more extensive atherosclerotic burden involving two or more arterial territories and is associated with a substantially higher risk of cardiovascular events and mortality than isolated lower-extremity disease [57]. Concomitant coronary, carotid, or aortic disease may remain clinically silent while retaining important prognostic implications [13,42,64]. Its recognition therefore contributes to systemic risk stratification and may identify patients who derive greater absolute benefit from intensive preventive treatment.

However, the high prevalence of subclinical disease does not justify indiscriminate anatomical screening of every vascular territory. Further coronary or cerebrovascular evaluation should be guided by symptoms, clinical findings, planned major vascular surgery, functional capacity, and the likelihood that diagnostic results would alter management. Regardless of additional testing, polyvascular involvement supports rigorous implementation of secondary prevention, whereas the selection and intensity of antithrombotic and lipid-lowering therapy should remain individualized according to the strength of the available evidence, competing bleeding risk, and patient characteristics [65].

### 6.6. High Bleeding Risk Versus High Ischemic Risk

Assessment of antithrombotic therapy in PAD is complicated by the frequent coexistence of ischemic and bleeding vulnerability. Advanced age, CKD, diabetes, anaemia, frailty, and polypharmacy may simultaneously increase the likelihood of cardiovascular or limb events and the risk of treatment-related hemorrhage [66]. These risks should therefore be evaluated concurrently rather than treated as mutually exclusive categories. Particular attention should be paid to previous major bleeding, renal function, haemoglobin levels, concomitant anticoagulant or antiplatelet therapy, and the presence of temporary procedural indications for treatment intensification.

Dual-pathway inhibition can reduce cardiovascular and limb events in appropriately selected patients with elevated ischemic risk [39]. Nevertheless, its expected absolute benefit must be weighed against bleeding susceptibility, especially in older or frail individuals and in those with renal impairment or a history of hemorrhage. Treatment intensity and duration should not be regarded as fixed decisions: they require reassessment after revascularization, changes in renal function or functional status, bleeding events, and completion of any procedural indication for dual-antiplatelet therapy [1]. Shared decision-making is particularly important when anticipated ischemic and bleeding risks are both high and the net clinical benefit remains uncertain.

### 6.7. Toward a Phenotype-Guided Treatment Model

The heterogeneity of PAD argues against a single therapeutic pathway for all patients. Asymptomatic PAD, intermittent claudication, chronic limb-threatening ischemia, ALI, and the post-revascularization state differ substantially in their immediate clinical priorities, expected event rates, and potential responses to treatment. A phenotype-guided approach should therefore integrate clinical presentation, previous limb events, polyvascular involvement, comorbidities, functional status, bleeding vulnerability, life expectancy, and patient preferences.

Within this model, treatment selection is based on the expected absolute benefit and harm for the individual patient rather than being inferred solely from biological mechanisms or relative treatment effects [67]. The principal therapeutic objective may vary between cardiovascular event prevention, limb preservation, symptom relief, wound healing, and functional improvement; in many patients, several objectives must be addressed simultaneously. Because both phenotype and treatment tolerance may change over time, the resulting management plan should be considered dynamic and periodically reassessed rather than applied as a fixed or formally validated treatment algorithm.

## 7. Contemporary Strategies to Reduce Mace and Male

PAD management requires the simultaneous and sustained control of thrombotic risk and modifiable cardiovascular risk factors. Antithrombotic therapy, intensive lipid lowering, blood pressure and diabetes management, smoking cessation, and structured physical activity should be implemented as complementary components of care rather than as isolated or sequential interventions. These measures remain necessary irrespective of symptomatic improvement or the technical success of revascularization, because restoration of limb perfusion does not eliminate the underlying systemic atherosclerotic burden. Treatment intensity should be individualized according to ischemic and bleeding risk, comorbidities, life expectancy, functional status, treatment tolerance, and patient preferences [1,2].

Therapeutic objectives should encompass cardiovascular event prevention, limb preservation, functional recovery, and maintenance of quality of life. These objectives are closely related but are not interchangeable. Antithrombotic and lipid-lowering therapies primarily target cardiovascular and thrombotic limb events, whereas exercise-based interventions predominantly improve walking performance, symptoms, and functional independence. Revascularization is directed toward symptom relief, wound healing, or limb salvage in appropriately selected patients but should be integrated with, rather than substituted for, long-term secondary prevention.

The expected absolute benefit of each intervention also varies according to the clinical phenotype. Patients with polyvascular disease, CLTI, previous lower-extremity revascularization or amputation, diabetes, CKD, or recurrent ischemic events generally have greater residual cardiovascular or limb risk and may derive greater benefit from treatment intensification, provided that bleeding risk and competing clinical conditions are acceptable. Polyvascular disease is particularly relevant because it identifies a population with a high systemic atherosclerotic burden and an increased incidence of both MACE and lower-extremity events [28].

Contemporary PAD care should therefore be structured as a coordinated and longitudinal strategy rather than as a succession of lesion-directed interventions. The following sections examine the principal therapeutic domains separately while distinguishing established guideline-supported treatments from PAD subgroup evidence, indirect extrapolation from broader cardiovascular populations, and emerging therapeutic approaches.

## 8. Antithrombotic Therapy

### 8.1. Antiplatelet Therapy in Symptomatic and Asymptomatic PAD

Antithrombotic therapy should be selected according to clinical phenotype, previous revascularization, concomitant vascular disease, and bleeding risk.

For symptomatic PAD, aspirin or clopidogrel is recommended as single antiplatelet therapy. CAPRIE supports clopidogrel as an effective option [59,68], whereas EUCLID found no superiority of ticagrelor over clopidogrel [27].

Evidence is less certain in asymptomatic PAD. Aspirin did not significantly reduce cardiovascular events in individuals with a low ABI but no established cardiovascular disease in the Aspirin for Asymptomatic Atherosclerosis trial [69]; treatment should therefore reflect overall cardiovascular risk rather than ABI status alone.

Routine long-term dual-antiplatelet therapy (DAPT) is not supported in stable PAD without a recent procedural indication, and full-dose anticoagulation should not be used solely for PAD in the absence of another indication.

### 8.2. Dual-Pathway Inhibition and Major Trial Evidence

In COMPASS, rivaroxaban 2.5 mg twice daily plus aspirin reduced MACE and MALE in stable PAD compared with aspirin alone, while increasing major bleeding [39]. After lower-extremity revascularization, the same regimen reduced ALI and major amputation in VOYAGER-PAD [70]. Dual-pathway inhibition should therefore be reserved for patients whose expected ischemic benefit exceeds their bleeding risk.

CAPRIE, EUCLID, CHARISMA, COMPASS, and VOYAGER-PAD define complementary clinical contexts. CHARISMA provided limited support for routine long-term DAPT in stable PAD because clopidogrel plus aspirin did not significantly reduce the primary cardiovascular endpoint and increased bleeding [71]. Contemporary guidelines accordingly distinguish single-antiplatelet therapy, dual-pathway inhibition, and time-limited post-procedural DAPT.

The principal randomized trials and clinically relevant subgroup analyses informing contemporary therapeutic strategies in PAD are summarized in Supplementary Table S3.

### 8.3. Antithrombotic Therapy After Revascularization

After endovascular intervention, DAPT with aspirin and a P2Y12 inhibitor may be used for a limited, procedure-dependent period. Prolonged DAPT has limited supporting evidence and should not be conflated with dual-pathway inhibition.

VOYAGER-PAD randomized 6564 patients undergoing lower-extremity revascularization to rivaroxaban 2.5 mg twice daily plus aspirin or aspirin alone. The combination reduced the composite of ALI, major amputation for vascular causes, myocardial infarction, ischemic stroke, or cardiovascular death, with the benefit driven particularly by a reduction in ALI. Although TIMI major bleeding was not significantly increased, ISTH major bleeding was more frequent with rivaroxaban [70].

Concomitant clopidogrel therapy requires particular consideration. A secondary publication from VOYAGER-PAD, specifically examining the influence of concomitant clopidogrel and distinct from the primary trial report, showed that the efficacy of rivaroxaban plus aspirin was preserved irrespective of clopidogrel use. However, longer-duration clopidogrel exposure was associated with a greater bleeding risk [72]. Unnecessarily prolonged triple antithrombotic therapy should therefore be avoided, and the continued indication for clopidogrel should be reassessed once the procedural indication for dual antiplatelet therapy has elapsed.

After surgical revascularization, therapy should reflect graft type, target, and systemic risk. CASPAR found no overall benefit from aspirin plus clopidogrel after below-knee bypass, although a prespecified prosthetic-graft subgroup appeared to benefit [73]. VOYAGER-PAD supports rivaroxaban plus aspirin after either surgical or endovascular revascularization when bleeding risk is acceptable [70].

### 8.4. Individualized Antithrombotic Selection

The absolute benefit of intensified therapy is greatest in patients with symptomatic disease, previous revascularization or ALI, polyvascular disease, diabetes, CKD, or recurrent events [1,2]. Previous major bleeding, active bleeding, severe anaemia, advanced renal dysfunction, frailty, or interacting therapies may favour a less intensive approach. Antithrombotic therapy should be reassessed as ischemic risk, bleeding susceptibility, renal function, medications, and procedural status change; the objective is net clinical benefit rather than maximal intensity.

## 9. Lipid-Lowering Therapy

### 9.1. High-Intensity Statins as Foundational Therapy

High-intensity or maximally tolerated statin therapy is the foundation of lipid management in PAD, with an aim of reducing LDL-C by at least 50%. Contemporary very-high-risk guidance supports an LDL-C goal near 55 mg/dL, while the 2024 ACC/AHA PAD guideline uses LDL-C ≥ 70 mg/dL on maximally tolerated statin therapy as a threshold for adding ezetimibe or a PCSK9 inhibitor. These values represent a treatment goal and an intensification threshold, respectively.

PAD-specific observational and pooled data associate statin therapy with lower mortality, MACE, and amputation, with greater benefit from higher-intensity regimens [74,75]. REACH similarly associated statin use with fewer cardiovascular and adverse limb outcomes [43,76].

Therapy should be optimized according to response, adherence, tolerance, and absolute risk. When adverse effects limit treatment, switching agents, dose adjustment, intermittent dosing, or combination with non-statin therapy may preserve clinically meaningful LDL-C reduction.

### 9.2. Ezetimibe in Residual LDL-C Elevation

Ezetimibe is a practical first-line non-statin option when LDL-C remains ≥70 mg/dL on maximally tolerated statin therapy or when statin intolerance limits dosing [43]. Although PAD-specific trial data are limited, IMPROVE-IT demonstrated cardiovascular benefit after acute coronary syndrome, with greater absolute benefit in high-risk polyvascular and diabetic subgroups [77].

### 9.3. PCSK9 Inhibitors and Cardiovascular and Limb Outcomes

PCSK9 inhibitors provide substantial additional LDL-C reduction. In FOURIER, evolocumab reduced MALE in the overall population (HR, 0.58; 95% CI, 0.38–0.88) [78]; among patients with symptomatic PAD, it reduced MACE (HR, 0.79; 95% CI, 0.66–0.94), while the MALE estimate was directionally favorable but not statistically significant [1].

ODYSSEY OUTCOMES likewise showed greater absolute benefit from alirocumab in polyvascular disease [79] and fewer PAD events overall (HR, 0.69; 95% CI, 0.54–0.89) [80]. In the subgroup with known PAD, MALE was reduced (HR, 0.59; 95% CI, 0.40–0.86) [1]. PCSK9 inhibition is therefore appropriate for selected high-risk patients above recommended LDL-C thresholds, while recognizing that limb outcomes were usually secondary or prespecified rather than primary endpoints [81].

### 9.4. Bempedoic Acid in Statin-Intolerant or High-Risk Patients

Bempedoic acid provides an additional oral LDL-C–lowering option for patients who are unable to tolerate guideline-directed statin therapy or who require further lipid reduction despite maximally tolerated lipid-lowering treatment. In CLEAR Outcomes, which enrolled 13,970 patients who were unable or unwilling to receive statin therapy because of adverse effects and who had established cardiovascular disease or were at high cardiovascular risk, bempedoic acid reduced the primary composite endpoint of cardiovascular death, nonfatal myocardial infarction, nonfatal stroke, or coronary revascularization compared with placebo (HR, 0.87) [82].

These findings support bempedoic acid as an alternative or adjunctive strategy for cardiovascular risk reduction, particularly in patients with statin intolerance. A prespecified subgroup analysis of CLEAR Outcomes also provided supportive evidence regarding limb outcomes among the 1624 participants with PAD. In this subgroup, bempedoic acid was associated with a 36% relative reduction in MALE as a first event (HR, 0.64; 95% CI, 0.44–0.93; *p* = 0.018) and a 45% reduction when total and recurrent events were considered (RR, 0.55; 95% CI, 0.35–0.85; *p* = 0.007) [83]. Although these adjudicated findings suggest a potential benefit on limb outcomes, they should be interpreted in the context of a subgroup analysis and should not be considered equivalent to evidence from a dedicated, adequately powered PAD trial. Accordingly, the results support—but do not definitively establish—a PAD-specific effect of bempedoic acid on MALE.

Bempedoic acid should not be regarded as a direct substitute for statins in patients who can tolerate guideline-directed statin therapy, which remains the foundation of lipid-lowering treatment in PAD. Rather, it may be considered in patients with complete or partial statin intolerance or as an adjunct when LDL-C targets are not achieved with maximally tolerated therapy. Combination therapy with ezetimibe may provide additional LDL-C reduction, particularly when only low-dose or intermittent statin treatment is tolerated.

Treatment selection should also account for the safety profile. In CLEAR Outcomes, bempedoic acid was associated with higher rates of hyperuricemia, gout, and cholelithiasis. These adverse effects warrant particular consideration in patients with pre-existing hyperuricemia, a history of gout, or other relevant predisposing conditions.

### 9.5. Future Directions in Lipid-Modifying Therapy

Lipoprotein(a) is a largely genetically determined risk factor associated with PAD-related events, although its relative effect is greater for myocardial infarction and aortic stenosis [84]. Antisense and small-interfering RNA therapies can markedly reduce circulating lipoprotein(a), but outcome trials have not yet established reductions in cardiovascular or limb events [85]. Current management therefore emphasizes measurement in appropriate patients and intensive control of established modifiable risk factors [84].

## 10. Metabolic and Cardiorenal Therapeutic Frontiers

### 10.1. Diabetes as a Driver of Cardiovascular and Limb Risk

Diabetes identifies a high-risk PAD phenotype characterized by accelerated atherosclerosis, endothelial and microvascular dysfunction, neuropathy, impaired wound healing, and increased risks of CLTI and amputation, particularly when CKD, smoking, infection, or poor glycemic control coexist [86]. Management should therefore extend beyond glucose targets to cardiovascular, renal, weight, and foot protection.

### 10.2. GLP-1 Receptor Agonists: Cardiovascular Benefit and Emerging Limb Signals

GLP-1RAs provide metabolic and cardiovascular benefits extending beyond glycemic control [87]. In patients with type 2 diabetes at high cardiovascular risk, LEADER demonstrated that liraglutide reduced the composite incidence of cardiovascular death, nonfatal myocardial infarction, or nonfatal stroke, while SUSTAIN-6 reported a similar cardiovascular benefit with semaglutide [88,89]. These findings primarily support the role of GLP-1RAs in reducing overall cardiovascular risk rather than establishing a therapeutic effect specifically directed toward PAD or limb-related outcomes.

The broader cardiovascular benefits of GLP-1RA therapy are clinically relevant to patients with PAD, who frequently have concomitant coronary, cerebrovascular, and polyvascular disease. Nevertheless, most GLP-1RA cardiovascular outcomes trials were not designed specifically for PAD, and the available PAD evidence is derived predominantly from subgroup, secondary, or exploratory analyses. Consequently, the evidence supporting a reduction in MACE is considerably stronger than that supporting prevention of MALE, revascularization, or amputation.

The PAD-specific STRIDE trial provided important evidence regarding functional outcomes. In patients with symptomatic PAD, intermittent claudication, and type 2 diabetes, once-weekly semaglutide improved maximum walking distance after 52 weeks, with additional favorable effects on symptoms and quality of life [90]. However, STRIDE was primarily designed to evaluate walking performance and was not adequately powered to establish reductions in cardiovascular or major adverse limb events. Its findings therefore support a functional benefit in selected patients with PAD but should not be interpreted as evidence of direct limb protection.

Evidence regarding MALE remains less definitive. Observational studies have associated GLP-1RA use with lower rates of MACE and MALE [91], whereas a 2026 meta-analysis restricted to randomized trials did not demonstrate a statistically significant reduction in revascularization or amputation [92]. Meta-analyses incorporating observational studies have suggested a potential reduction in limb events; however, substantial heterogeneity, residual confounding, and the very low certainty of the observational evidence limit causal inference [93]. Dedicated randomized trials with prespecified and adequately powered limb endpoints are therefore required before a PAD-specific protective effect can be established.

The cardiovascular benefits of GLP-1RA therapy also extend beyond patients with diabetes. In SELECT, semaglutide 2.4 mg reduced the risk of cardiovascular death, nonfatal myocardial infarction, or nonfatal stroke by 20% in patients with established cardiovascular disease and overweight or obesity without diabetes [94]. In patients with PAD and obesity, weight reduction may additionally facilitate mobility and participation in exercise-based rehabilitation, although this potential benefit remains indirect and should be balanced against nutritional status, sarcopenia, and frailty, particularly in patients with advanced disease.

Accordingly, GLP-1RAs should currently be prescribed principally on the basis of their established metabolic, weight-management, and cardiovascular indications. Their potential benefits on walking function and limb outcomes in PAD remain promising but should be regarded as complementary and still-emerging evidence rather than as established PAD-specific therapeutic effects.

### 10.3. SGLT2 Inhibitors: Cardiovascular and Renal Protection in PAD

SGLT2 inhibitors provide cardiovascular and renal benefits that extend beyond glucose lowering. EMPA-REG OUTCOME demonstrated that empagliflozin reduced major cardiovascular events and cardiovascular mortality in patients with type 2 diabetes and established cardiovascular disease, while subsequent analyses showed substantial attenuation of kidney disease progression [95,96]. DAPA-CKD further demonstrated that dapagliflozin reduced the risk of kidney failure, sustained decline in estimated glomerular filtration rate, and cardiovascular or renal death in patients with CKD, including those without diabetes [97]. These findings primarily establish the cardiorenal efficacy of SGLT2 inhibitors and should not be interpreted as evidence of a PAD-specific therapeutic effect.

The cardiorenal benefits of SGLT2 inhibitors are nevertheless clinically relevant to patients with PAD, who frequently have concomitant diabetes, CKD, heart failure, and polyvascular disease. CKD is an important amplifier of cardiovascular and limb risk, and preservation of renal function may indirectly improve the long-term prognosis of patients with PAD. However, this potential indirect benefit does not establish a direct reduction in MALE, limb revascularization, or amputation.

The relationship between SGLT2 inhibitors and lower-extremity amputation warrants particular consideration because of the safety signal observed with canagliflozin in CANVAS, in which amputations occurred approximately twice as frequently as with placebo [98]. Subsequent randomized trials have not consistently reproduced this finding with canagliflozin or other agents in the class, and meta-analytic evidence has not demonstrated a statistically significant class-wide increase in amputation risk, although heterogeneity among agents, populations, and endpoint definitions remains [99]. A prespecified PAD analysis of DECLARE–TIMI 58 showed that dapagliflozin provided cardiovascular and renal benefits consistent with those observed in the overall trial population, without significantly reducing MACE or PAD-specific limb events. Importantly, dapagliflozin was not associated with a statistically significant increase in ischemic limb events or amputations, although the limited size of the PAD subgroup and the wide confidence intervals warrant cautious interpretation. These findings support the cardiovascular and renal use of dapagliflozin in patients with PAD while providing no definitive evidence of direct limb protection [100].

Current evidence therefore does not support attributing a uniform increase in amputation risk to the entire SGLT2 inhibitor class. Nevertheless, careful foot surveillance and individualized risk assessment remain appropriate, particularly in patients with active ulceration, infection, previous amputation, severe ischemia, or chronic limb-threatening ischemia.

Evidence supporting PAD-specific efficacy remains substantially less developed than that supporting cardiovascular, heart-failure, and renal outcomes. Most available PAD data originate from subgroup or secondary analyses of broader cardiovascular outcomes trials, and a consistent reduction in MALE has not been established. SGLT2 inhibitors should therefore be selected primarily according to their established indications for heart failure, CKD, diabetes, and overall cardiovascular risk reduction. Their use in patients with PAD may provide important systemic cardiorenal protection, but they should not currently be regarded as therapies specifically targeting limb-related outcomes [101].

### 10.4. Obesity and Precision Cardiometabolic Prevention

Obesity and insulin resistance interact with inflammation, dyslipidemia, hypertension, and functional limitation. Assessment should include central adiposity, glycemic status, renal function, physical activity, and nutritional status rather than BMI alone [102], SELECT demonstrated cardiovascular benefit from semaglutide in established cardiovascular disease with overweight or obesity, although weight loss in advanced PAD must be balanced against sarcopenia and frailty [94].

Metabolic phenotyping can identify established diabetes, obesity, CKD, or heart-failure indications for treatment, but insulin resistance alone does not currently define a PAD-specific pharmacological strategy.

The expanding evidence for GLP-1RAs and SGLT2 inhibitors supports a transition from glucose-centered treatment toward phenotype-driven cardiometabolic prevention. In patients with type 2 diabetes and established atherosclerotic cardiovascular disease, GLP-1RAs may be particularly useful when reduction of atherosclerotic risk and weight management are priorities, whereas SGLT2 inhibitors are especially relevant in the presence of CKD or heart failure. Patients with obesity and established cardiovascular disease may also benefit from intensive weight-management strategies, including GLP-1RA-based therapy.

FLOW showed renal benefit from semaglutide in type 2 diabetes and CKD [103], with prespecified analyses suggesting persistence of benefit during concomitant SGLT2 inhibition; however, the subgroup was small and not PAD-specific, and additive limb benefit remains uncertain [104].

Cardiometabolic agents should complement, not replace, antithrombotic therapy, lipid lowering, blood pressure control, smoking cessation, exercise, and limb surveillance. Dedicated PAD trials powered for MALE, amputation, revascularization, and function remain limited; STRIDE provides important functional evidence but not definitive limb-event protection [90].

Thus, GLP-1 receptor agonists and SGLT2 inhibitors should primarily be selected for established metabolic, cardiovascular, renal, heart-failure, or weight-management indications; direct limb protection remains emerging evidence.

The principal randomized trials and clinically relevant subgroup analyses informing metabolic and exercise-based strategies in PAD are summarized in Supplementary Table S4.

## 11. Blood Pressure Control, Smoking Cessation, and Global Risk-Factor Management

Hypertension is common in PAD and should be managed as part of comprehensive cardiovascular prevention.

A blood pressure target below 130/80 mmHg is generally recommended when safely tolerated. More intensive systolic lowering may benefit selected high-risk patients, but treatment should account for age, frailty, renal function, orthostatic symptoms, and functional status [82,105]. ACCORD-BP did not significantly reduce its primary composite with a systolic target below 120 mmHg, whereas the adequately powered BPROAD trial reported an approximately 21% reduction in major cardiovascular events [106,107].

Excessive lowering should be avoided when it causes hypotension or functional impairment. Antihypertensive selection should reflect the cardiorenal phenotype; beta-blockers need not be withheld when otherwise indicated [1,2], and ramipril reduced cardiovascular events in high-risk patients, including those with peripheral vascular disease, in HOPE [108].

Smoking is among the strongest modifiable determinants of PAD progression. Continued smoking after bypass is associated with approximately threefold greater graft failure, whereas cessation improves patency [109]. Smoking cessation is also associated with lower mortality and improved amputation-free survival [110], fewer adverse outcomes after revascularization [111], and lower major-amputation risk in CLTI [112]. Repeated counseling, behavioral support, and pharmacotherapy should be integrated into longitudinal care.

Diabetes care should combine individualized glycemic targets with therapies selected for cardiovascular and renal benefit, weight management, and prevention of foot complications. Lower HbA1c variability is associated with better outcomes in observational PAD cohorts [113], but glucose lowering alone is insufficient. Regular foot assessment, patient education, prompt treatment of ulceration or infection, and monitoring of estimated glomerular filtration rate and urinary albumin-to-creatinine ratio are essential [114].

Lifestyle care should promote a heart-healthy diet, daily physical activity, appropriate weight management, and structured exercise as detailed in Section 12. Recommendations should be adapted to renal function, nutritional status, frailty, and socioeconomic circumstances.

Long-term prevention also requires attention to adherence, health literacy, psychosocial barriers, and access to care. Periodic reassessment of blood pressure, lipids, glycemic status, smoking, renal function, activity, weight, and new cardiovascular or limb symptoms should replace episodic, procedure-centered care.

Multidisciplinary coordination among vascular, primary-care, cardiology, diabetes, pharmacy, nutrition, podiatry, and exercise teams can support simultaneous and durable control of modifiable risk factors.

## 12. Exercise Therapy and Functional Restoration

Structured exercise is first-line therapy for chronic symptomatic PAD because it improves walking performance, functional status, and quality of life. Supervised exercise therapy (SET) commonly uses intermittent walking at least three times weekly for at least 12 weeks, progressing to moderate or greater claudication discomfort according to tolerance [115].

Benefits reflect skeletal-muscle, mitochondrial, endothelial, and metabolic adaptations and may occur without measurable improvement in resting limb perfusion [111,112]. In CLEVER, SET improved treadmill performance more than primary stenting at six months, although stenting produced greater gains in some disease-specific quality-of-life measures [113]. Exercise should not delay urgent vascular evaluation in CLTI or tissue loss.

When SET is inaccessible, structured home- or community-based exercise is an evidence-based alternative. Effective programs prescribe frequency, intensity, duration, and progression and may incorporate coaching, monitoring, and behavioral support; unstructured advice to simply walk more is not equivalent.

In LITE, low-intensity walking that avoided ischemic symptoms was inferior to higher-intensity walking and did not outperform control for six-minute walk distance [114]. GOALS demonstrated sustained functional improvement with a behaviorally supported home program [115]. An individual-participant meta-analysis found larger treadmill gains with SET but greater six-minute-walk and patient-reported improvements with home-based exercise [116], partly reflecting training to the tested modality [117].

SET and structured home programs should therefore be considered complementary, allowing transition to a sustainable long-term regimen while maintaining adequate intensity.

Response should be assessed with clinically meaningful outcomes, including pain-free and maximal walking distance, six-minute walk distance, daily activity, patient-reported walking impairment, and quality of life [118]. ABI and imaging define hemodynamic and anatomical disease but do not capture functional benefit.

Exercise has established benefits for symptoms, mobility, and quality of life, whereas evidence for reducing MACE or MALE remains limited. It should therefore complement rather than replace medical prevention.

Exercise may improve skeletal-muscle, endothelial, mitochondrial, and cardiometabolic function beyond symptom relief, but trials have generally not been powered for myocardial infarction, stroke, ALI, or amputation. It remains valuable before and after revascularization to address deconditioning and persistent functional limitation, with long-term adherence supported through structured home or community programs.

## 13. Revascularization and Post-Procedural Secondary Prevention

Revascularization should be guided by the clinical objective rather than stenosis alone. In functionally limiting claudication, it is considered when symptoms persist despite guideline-directed therapy and structured exercise; in CLTI, it aims to restore perfusion, promote wound healing, relieve ischemic pain, and preserve a functional limb [119]. It does not independently reduce systemic cardiovascular risk and must remain integrated with secondary prevention.

Endovascular and surgical strategies are complementary. Selection should consider anatomy, conduit availability, comorbidity, procedural risk, life expectancy, previous interventions, and patient preference. Endovascular treatment generally has a lower initial procedural burden, whereas autogenous-vein bypass remains important in suitable patients.

In BEST-CLI, bypass reduced MALE or death compared with endovascular treatment when an adequate great saphenous vein was available, but not when it was unavailable [120]. BASIL-2 found better amputation-free survival with an endovascular-first strategy for infrapopliteal disease, largely because of lower mortality [121]. These differing results support individualized rather than universal procedure selection.

After lower-extremity revascularization, recurrent ALI, revascularization, amputation, MACE, and bleeding remain important. In VOYAGER-PAD, rivaroxaban 2.5 mg twice daily plus aspirin reduced the composite cardiovascular and limb endpoint (HR, 0.85; 95% CI, 0.76–0.96) but increased ISTH major bleeding (HR, 1.42; 95% CI, 1.10–1.84) [70]. Benefit was consistent after endovascular and surgical procedures [122], with a 33% reduction in ALI [123].

Dual-pathway inhibition should therefore be considered in appropriate patients without excessive bleeding risk or another indication for full-dose anticoagulation, as part of comprehensive post-procedural prevention.

Surveillance should be tailored to the procedure, anatomical territory, clinical status, and new symptoms. ABI is useful but may be falsely elevated by medial calcification; toe pressures or other perfusion measures can provide complementary information in diabetes, CKD, or CLTI.

Duplex ultrasound is particularly useful for bypass grafts and selected endovascular interventions. Current ACC/AHA and ESC guidance supports longitudinal clinical follow-up, ABI and duplex imaging for new signs or symptoms, and selective scheduled surveillance after infrainguinal vein bypass or endovascular procedures. Patients should be taught to recognize sudden pain, pallor, coldness, sensory loss, or weakness suggestive of ALI.

Long-term follow-up should evaluate recovery, symptoms, wound healing, recurrent ischemia, functional capacity, adherence, smoking, blood pressure, lipids, glycemic control, and renal function. CLTI requires closer multidisciplinary monitoring of perfusion, infection, pressure relief, wound healing, and foot care.

Because restored perfusion does not necessarily normalize walking capacity, exercise and rehabilitation should accompany repeated optimization of antithrombotic, lipid-lowering, blood pressure, diabetes, and smoking interventions. Follow-up should therefore target recurrent MACE and MALE, ambulation, independence, and quality of life rather than graft or device patency alone.

## 14. Personalized Four-Domain Clinical Framework

Contemporary PAD management requires individualized assessment of cardiovascular and limb risk rather than a uniform treatment strategy. The risks of MACE and MALE vary according to clinical presentation, previous lower-extremity revascularization or amputation, polyvascular disease, concomitant CAD, comorbidities, and treatment history. Therapeutic decisions should therefore be guided by the patient’s absolute ischemic risk, bleeding risk, modifiable residual risk, and clinical objectives. These variables should also be interpreted dynamically, because thrombotic and limb risk are not constant over time and are particularly elevated during the early period after acute limb ischemia or lower-extremity revascularization [2].

The initial assessment should characterize limb and systemic vascular risk separately. CLTI, previous lower-extremity revascularization or amputation, acute or recurrent limb ischemia, and complex lower-extremity disease identify patients at particularly high risk of MALE. Polyvascular disease, concomitant CAD, diabetes, CKD, heart failure, and previous cardiovascular events further increase the risk of MACE and may increase the absolute benefit derived from intensified preventive therapy. Conversely, patients without concomitant CAD, previous peripheral revascularization, or other major MACE or MALE risk features may derive less absolute benefit from combination antithrombotic regimens; in this lower-risk phenotype, single antiplatelet therapy generally represents an appropriate initial strategy [3].

Concomitant indications for antithrombotic therapy require specific consideration. In patients with CAD requiring DAPT, the choice and duration of treatment should primarily follow the coronary indication while also accounting for limb risk; ticagrelor-based DAPT may provide additional protection against limb events in appropriately selected patients, although its potential benefit must be balanced against bleeding risk. Patients with atrial fibrillation or another established indication for full-dose anticoagulation should continue anticoagulation at the evidence-based therapeutic dose rather than receive a reduced vascular dose intended for PAD prevention. Additional antiplatelet therapy should be limited to situations in which there is a clear coronary or peripheral procedural indication and should be reassessed once that indication has elapsed [3].

Bleeding risk should be assessed concurrently, particularly before antithrombotic intensification. Advanced age, previous major bleeding, anaemia, thrombocytopenia, renal dysfunction, low body weight, frailty, and concomitant antithrombotic or anticoagulant therapy may reduce the net clinical benefit of more intensive treatment. Structured instruments such as the Academic Research Consortium High Bleeding Risk criteria may be used as adjuncts to clinical assessment, although they should be interpreted contextually and should not replace individualized clinical judgment. Ischemic and bleeding risks should therefore be considered jointly, and more intensive antithrombotic therapy should be pursued only when the anticipated ischemic benefit outweighs the bleeding hazard [3]. Because therapeutic priorities differ substantially according to clinical presentation, phenotype-specific management recommendations for asymptomatic PAD, intermittent claudication, CLTI, acute limb ischemia, and post-revascularization care are summarized in Table 1.

The assessment should subsequently identify modifiable residual risk, including uncontrolled atherogenic lipid levels, hypertension, diabetes, smoking, physical inactivity, obesity, inadequate foot care, and suboptimal treatment adherence. Management should address these factors comprehensively rather than sequentially, integrating antithrombotic therapy, lipid lowering, blood pressure and diabetes management, smoking cessation, exercise, and preventive foot care according to individual needs [5]. Practical treatment targets include an LDL-C reduction of at least 50% from baseline. The recommended absolute LDL-C goal differs among guidelines: European and selected diabetes or consensus documents support a target of <55 mg/dL in very-high-risk patients, whereas the ACC/AHA PAD guideline supports intensification when LDL-C remains ≥70 mg/dL. Blood pressure should generally be reduced to <130/80 mm Hg when safely tolerated. In patients with diabetes, SGLT2 inhibitors and GLP-1 receptor agonists should be prioritized according to their established cardiovascular, renal, metabolic, and weight-management indications, while recognizing that their direct effects on PAD-specific limb outcomes remain less certain [8,9].

A practical four-domain framework for individualized therapeutic decision-making can therefore be organized around four questions (Figure 2): (1) What is the patient’s current MACE and MALE risk, and how does this risk vary over time? (2) What is the bleeding risk, and does it permit treatment intensification? (3) Which modifiable residual-risk factors and evidence-based therapeutic targets remain inadequately controlled? and (4) Is the principal therapeutic objective cardiovascular risk reduction, limb preservation, functional improvement, or a combination of these? This framework is intended to support rather than replace clinical judgment.

It allows the composition and intensity of treatment to be aligned with the anticipated absolute benefit, safety profile, functional status, and patient priorities, with planned reassessment and potential de-escalation as the clinical condition and risk profile evolve.

## 15. Gaps in Evidence and Future Directions

### 15.1. Evidence Generalizability and Representation

PAD trials differ from routine practice in age, comorbidity, anatomical complexity, frailty, phenotype, and access to care. Asymptomatic PAD, claudication, CLTI, ALI, and post-revascularization disease have distinct risks and objectives, limiting uniform extrapolation. Patients with advanced CKD, heart failure, frailty, diabetes, previous amputation, or severe functional impairment are often underrepresented; the CLEVER-PORTRAIT comparison illustrates this external-validity gap [124].

Women, older adults, and racial and ethnic minority groups remain underrepresented in PAD trials [125]; women accounted for approximately 28% of participants in one systematic review, with still lower representation of several minority populations [126]. Because demographic, socioeconomic, and access factors influence presentation and outcomes [127], future studies require broader eligibility, inclusive recruitment, and systematic phenotype characterization. Community ABI-based recruitment offers one feasible approach [128].

### 15.2. Standardized and Patient-Centered Outcomes

Heterogeneous MALE definitions impede comparison across trials. The Peripheral Academic Research Consortium provides a framework for standardized clinical, anatomical, procedural, imaging, and physiological outcomes [129], but major amputation, ALI, progression to CLTI, wound healing, ambulation, and functional recovery should be reported separately from repeat revascularization or patency. Event timing, severity, recurrence, MACE, mortality, bleeding, and quality of life should also be captured.

### 15.3. Emerging Risk Stratification and Precision Medicine

Biomarkers, advanced imaging, digital phenotyping, and artificial intelligence may refine risk assessment but require evidence of incremental and actionable clinical value. A six-protein model predicted two-year MALE with an area under the curve near 0.84 but requires external validation [130]. AI applied to Doppler waveforms and procedural data has also shown prognostic potential [131,132,133,134].

The 2026 American Heart Association advisory identifies opportunities across screening, imaging, prediction, and treatment planning while emphasizing validation, bias, explainability, data quality, regulation, and implementation [133]. Future models should demonstrate calibration, fairness, clinical utility, and patient benefit across representative populations.

Precision vascular medicine should identify clinically meaningful phenotypes rather than imply a unique treatment for every patient. Risk assessment should integrate anatomy, ischemic and bleeding risk, diabetes, CKD, frailty, tissue loss, polyvascular disease, function, previous events, and patient goals. Established antithrombotic therapy, lipid lowering, blood pressure and diabetes management, smoking cessation, exercise, foot care, and timely revascularization remain foundational [43]; emerging biomarkers, imaging, and digital tools should be adopted only after reproducible clinical utility is demonstrated [135,136,137,138].

## 16. Discussion

Contemporary evidence indicates that PAD should no longer be regarded as a localized arterial disorder, but rather as a manifestation of systemic atherosclerosis characterized by frequent multiterritory vascular involvement. Routine screening for coronary artery disease (CAD) in patients with newly diagnosed, clinically stable PAD is not currently recommended, because there is no evidence that the systematic detection of asymptomatic CAD improves clinical outcomes or warrants coronary revascularization in the absence of conventional indications. Irrespective of cardiac testing, most patients with PAD should receive intensive preventive therapy, including high-intensity or maximally tolerated lipid-lowering treatment, appropriate antithrombotic therapy, rigorous control of modifiable risk factors, and smoking cessation, because PAD itself identifies a population at high or very high cardiovascular risk. Further investigation for CAD should therefore be individualized and reserved for circumstances in which the findings are likely to modify clinical management, including symptoms or signs suggestive of myocardial ischemia or heart failure, relevant electrocardiographic or biomarker abnormalities, and preoperative assessment before major vascular surgery in patients with poor functional capacity or an elevated clinical-risk profile. When noninvasive evaluation is indicated, stress imaging or coronary computed tomography angiography may generally be preferred to invasive coronary angiography, unless the latter is otherwise clinically warranted. A particularly high-risk polyvascular phenotype, including concomitant lower-extremity PAD and carotid disease, is associated with a high prevalence of occult CAD and may strengthen the rationale for selective investigation; however, multiterritory atherosclerosis alone should not be considered an indication for universal coronary screening. Diagnostic evaluation should therefore be guided by symptoms, perioperative risk, functional capacity, and the likelihood that its findings will alter management, rather than by the prevalence of asymptomatic CAD alone.

The systemic nature of PAD is supported by pathophysiological features shared with coronary and cerebrovascular atherosclerosis, including endothelial dysfunction, oxidative stress, inflammation, and a prothrombotic state. Clinical studies have reported additional vascular-territory involvement in approximately 40% to 71% of patients with symptomatic PAD [27,29]. The extent of polyvascular involvement is associated with progressively higher cardiovascular risk and a greater incidence of myocardial infarction, ischemic stroke, and cardiovascular death. Limb risk also varies substantially according to clinical phenotype: five-year rates of major adverse limb events (MALE) are approximately 5–10% among patients with intermittent claudication and increase markedly in those with CLTI [35]. PAD is likewise associated with a significantly higher incidence of major adverse cardiovascular events (MACE; RR, 1.73) [2]. Thus, PAD should be interpreted both as a manifestation of systemic atherosclerotic burden and as an independent marker of adverse cardiovascular, limb, and mortality outcomes.

This heterogeneity supports a phenotype-guided therapeutic approach. Acute limb ischemia requires emergency assessment and timely revascularization, whereas CLTI warrants prompt multidisciplinary evaluation and a limb-salvage strategy that includes revascularization whenever technically feasible and clinically appropriate. In chronic PAD, management should combine limb-directed interventions with comprehensive cardiovascular risk reduction. Single-antiplatelet therapy with either clopidogrel or aspirin remains a guideline-supported option for stable symptomatic PAD [68]. Dual-pathway inhibition with low-dose rivaroxaban and aspirin provides additional cardiovascular and limb protection in appropriately selected patients at elevated ischemic risk, including those with previous lower-extremity revascularization, provided that bleeding risk is not prohibitive [39]. Decisions regarding intensified antithrombotic therapy should therefore integrate ischemic and hemorrhagic risk, comorbidities, renal function, frailty, previous bleeding, and the patient’s overall therapeutic objectives.

Intensive lipid lowering is another cornerstone of PAD management. High-intensity or maximally tolerated statin therapy should be used as first-line treatment, with the addition of ezetimibe, PCSK9 inhibitors, or bempedoic acid when clinically appropriate to achieve the recommended LDL-C reduction and, in very-high-risk patients, an LDL-C target below 55 mg/dL. Pharmacological treatment should be integrated with smoking cessation, structured physical activity, dietary and weight management, and optimal control of diabetes, hypertension, and CKD [78]. Therapies targeting specific inflammatory pathways remain under investigation. Although they may ultimately contribute to the reduction of systemic atherosclerotic risk, current evidence is insufficient to support their routine use for PAD-specific prevention of MACE or MALE.

A practical risk-based framework should therefore relate treatment intensity to the patient’s clinical phenotype, systemic atherosclerotic burden, absolute ischemic risk, bleeding risk, and therapeutic goals. However, the evidence supporting different interventions is not uniform. Limb outcomes have frequently been evaluated as secondary, prespecified, subgroup, or exploratory endpoints rather than as primary outcomes in trials specifically powered for PAD. Consequently, the evidence base for MALE prevention remains less extensive than that for MACE, limiting the certainty with which some PAD-specific treatment effects can be estimated and underscoring the need for dedicated randomized trials with standardized cardiovascular, limb, bleeding, and functional outcomes.

## 17. Conclusions

PAD is a systemic, heterogeneous atherosclerotic disease in which cardiovascular, limb, bleeding, metabolic, and functional risks must be assessed together. Established management combines antithrombotic therapy, intensive lipid lowering, blood pressure and diabetes care, smoking cessation, structured exercise, foot care, and revascularization when clinically indicated.

Future studies should recruit representative populations, standardize cardiovascular, limb, bleeding, and functional endpoints, and evaluate integrated treatment pathways over longer follow-up. The objective is sustained reduction of MACE and MALE while preserving limb function, independence, quality of life, and equity of care.

## Figures and Tables

**Figure 1:**
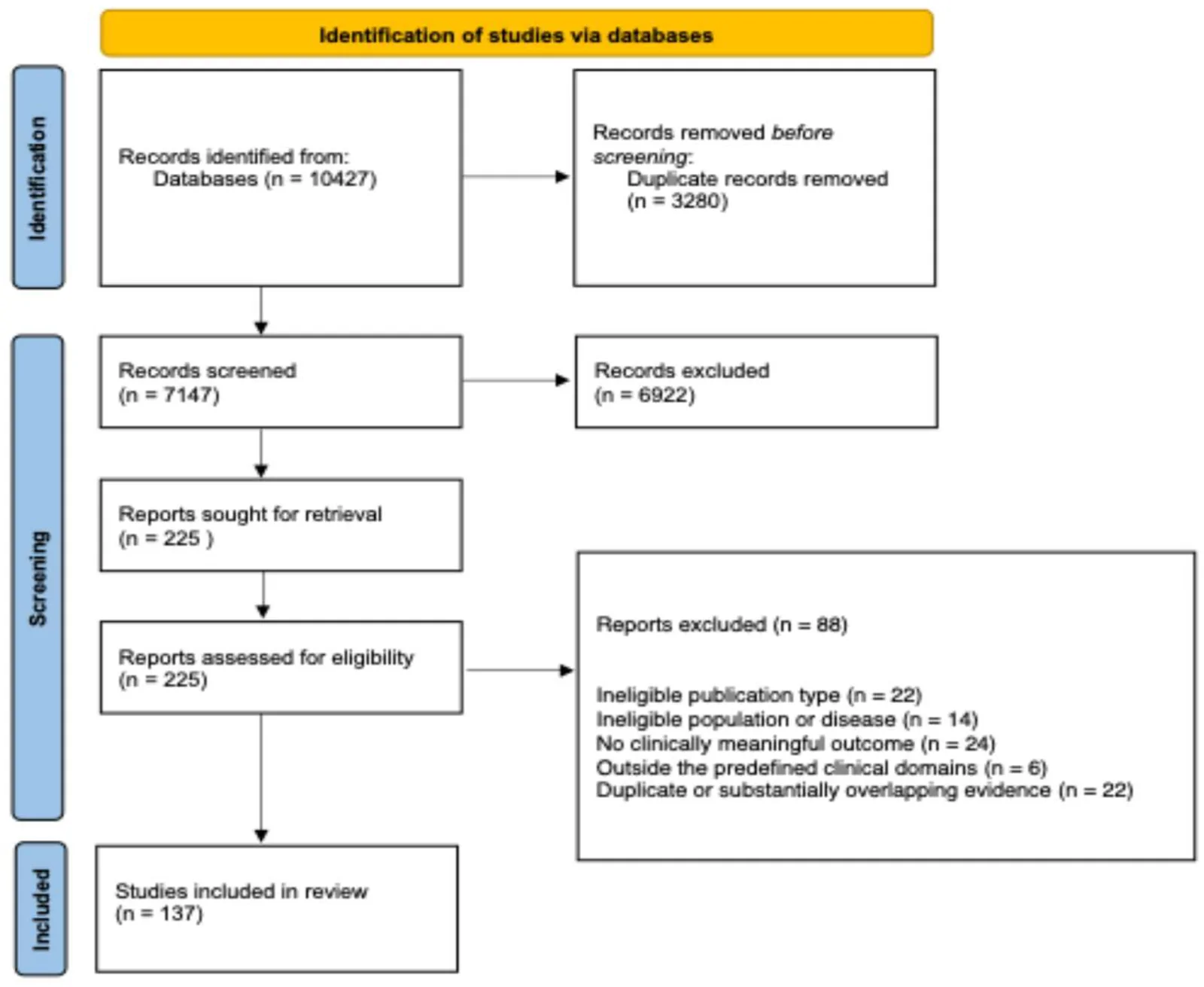
PRISMA 2020 flow diagram of the literature search and study selection process. Source: Ref. [4]. This work is licensed under CC BY 4.0. To view a copy of this license, visit https://creativecommons.org/licenses/by/4.0/. (accessed on 4 September 2026).

**Figure 2:**
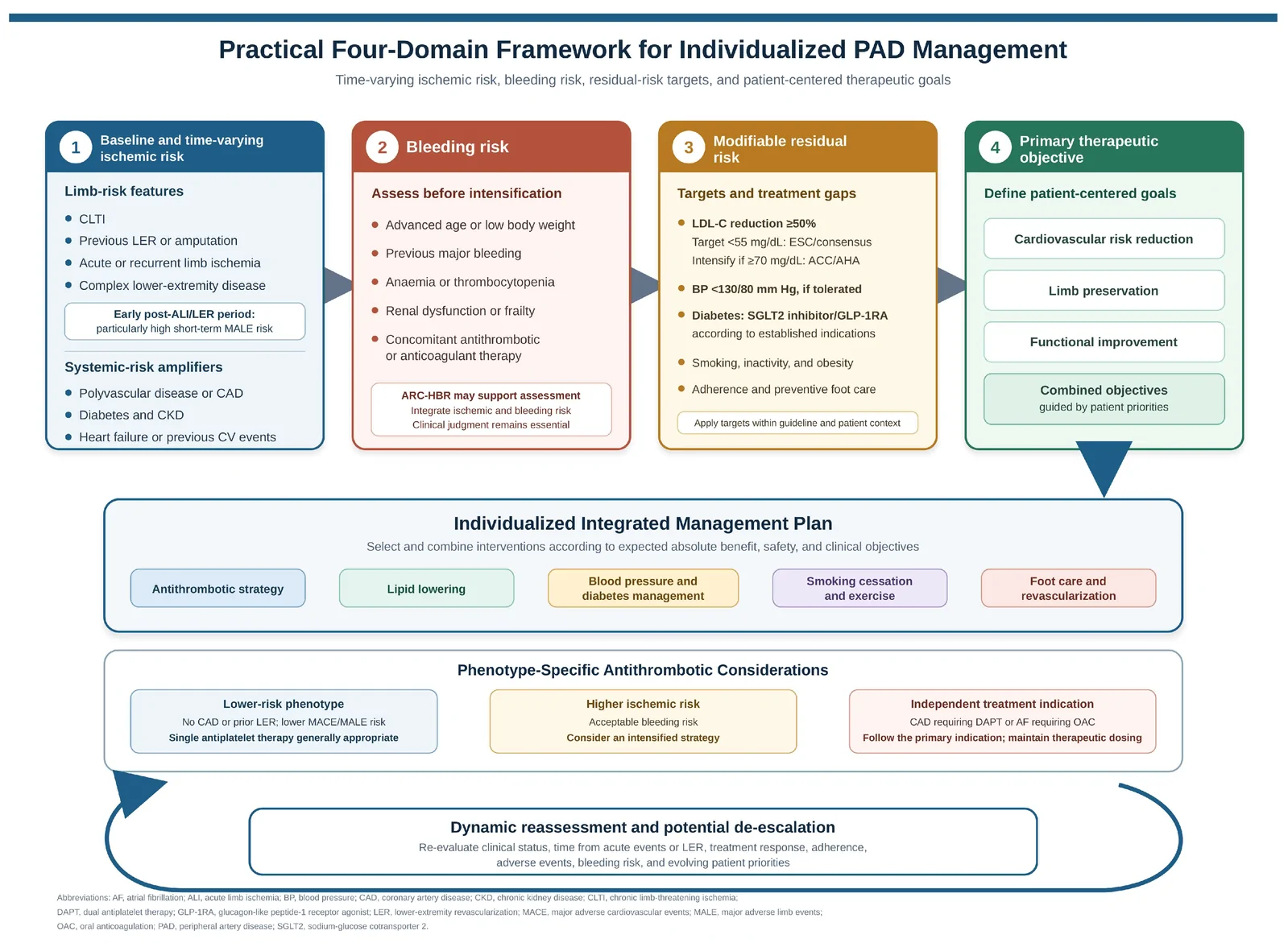
Practical Four Domain framework for individualized PAD management.

**Table 1:** Therapeutic priorities in PAD according to clinical presentation.

Clinical Phenotype	Principal Therapeutic Priorities
Asymptomatic PAD	Intensive cardiovascular prevention; exercise and risk-factor control; no revascularization solely to prevent disease progression
Intermittent claudication	Guideline-directed medical therapy, structured exercise, symptom-directed pharmacotherapy and selective revascularization for persistent functionally limiting symptoms
CLTI	Urgent multidisciplinary vascular assessment, limb-salvage revascularization when feasible, wound and infection management, foot care and intensive systemic prevention
Acute limb ischemia	Emergency vascular assessment, immediate anticoagulation when not contraindicated and urgent reperfusion according to limb viability
Post-revascularization PAD	Procedure-appropriate antithrombotic therapy, consideration of dual-pathway inhibition when bleeding risk is acceptable, surveillance, wound care and reassessment over time

## Data Availability

No new data were created or analyzed in this study. Data sharing is not applicable to this article.

## Supplementary Materials

The following supporting information can be downloaded at: https://www.mdpi.com/article/10.3390/medicina62101905/s1, Supplementary Table S1. Database-specific search strategies used for the literature review; Supplementary Table S2. Concept-driven subdivision of the global search strategy; Supplementary Table S3. Principal clinical evidence informing therapeutic strategies in peripheral artery disease-Panel A. Study design, population, intervention, and endpoint definitions; Supplementary Table S3. Principal clinical evidence informing therapeutic strategies in peripheral artery disease continued-Panel B. Clinical outcomes and interpretive limitations; Supplementary Table S4. Clinical evidence supporting metabolic and exercise-based strategies in peripheral artery disease-Panel A1. Metabolic strategies: study design, population, intervention, and endpoint definitions-Panel A2. Metabolic strategies: clinical outcomes and interpretive limitations-Panel B1. Exercise-based strategies: study design, population, intervention, and endpoint definitions-Panel B2. Exercise-based strategies: clinical outcomes and interpretive limitations.

## Abbreviations

The following abbreviations are used in this manuscript: ABI: Ankle-Brachial Index; ACC/AHA: American College of Cardiology/American Heart Association; ALI: Acute limb ischemia; BP: blood pressure; CAD: Coronary artery disease; CCD: Chronic Coronary Syndrome; CKD: chronic kidney disease; CLTI: chronic limb-threatening ischemia; DAPT: dual-antiplatelet therapy; DUS: Duplex ultrasound; GLP-1: glucagon-like peptide-1; GLP-1RAs: GLP-1 receptor agonists; HR: Hazard ratio; hs-CRP: high-sensitive C-reactive protein; IC: intermittent claudication; IL-6: interleukin-6; Lp(a): Lipoprotein(a); LDL: low-density lipoprotein; MACE: major adverse cardiovascular events; MALE: major adverse limb events; PAD: Peripheral artery disease; SGLT2: sodium-glucose cotransporter-2; SET: Supervised exercise therapy; TBI: Toe-Brachial Index.
