# Supplementary material for: PAD as a Marker of Systemic Atherosclerosis: A Comprehensive Review of Residual Risk and Contemporary Strategies for MACE and MALE Reduction
Source: Medicina (Kaunas). 2026 Oct 2;62(10):1905. doi: 10.3390/medicina62101905 (PMC13707036; doi:10.3390/medicina62101905)
Supplement: Supplementary file 1 [file medicina-62-01905-s001.zip › Supplementary Table S1.pdf]

Database	Search strategy
PubMed/MEDLINE	(("peripheral artery disease"[Title/Abstract] OR "peripheral arterial disease"[Title/Abstract] OR PAD[Title/Abstract]) AND ("systemic atherosclerosis"[Title/Abstract] OR "polyvascular disease"[Title/Abstract] OR "polyvascular atherosclerotic disease"[Title/Abstract] OR "multivessel atherosclerosis"[Title/Abstract] OR "major adverse cardiovascular events"[Title/Abstract] OR "major adverse cardiac events"[Title/Abstract] OR MACE[Title/Abstract] OR "major adverse limb events"[Title/Abstract] OR MALE[Title/Abstract] OR "antithrombotic therapy"[Title/Abstract] OR antithrombotic*[Title/Abstract] OR anticoagul*[Title/Abstract] OR antiplatelet*[Title/Abstract] OR rivaroxaban[Title/Abstract] OR aspirin[Title/Abstract] OR "dual pathway inhibition"[Title/Abstract] OR "PCSK9 inhibitor*"[Title/Abstract] OR evolocumab[Title/Abstract] OR alirocumab[Title/Abstract] OR "bempedoic acid"[Title/Abstract] OR "GLP-1 receptor agonist*"[Title/Abstract] OR semaglutide[Title/Abstract] OR liraglutide[Title/Abstract] OR dulaglutide[Title/Abstract] OR "SGLT2 inhibitor*"[Title/Abstract] OR empagliflozin[Title/Abstract] OR dapagliflozin[Title/Abstract] OR canagliflozin[Title/Abstract] OR "exercise therapy"[Title/Abstract] OR "supervised exercise"[Title/Abstract] OR "supervised exercise therapy"[Title/Abstract] OR SET[Title/Abstract] OR rehabilitation[Title/Abstract] OR "risk stratification"[Title/Abstract] OR "residual risk"[Title/Abstract] OR prognosis[Title/Abstract] OR prognostic[Title/Abstract] OR prediction[Title/Abstract] OR predictor*[Title/Abstract]))

Database	Search strategy
Embase	('peripheral artery disease':ti,ab OR 'peripheral arterial disease':ti,ab OR PAD:ti,ab) AND ('systemic atherosclerosis':ti,ab OR 'polyvascular disease':ti,ab OR 'polyvascular atherosclerotic disease':ti,ab OR 'multivessel atherosclerosis':ti,ab OR 'major adverse cardiovascular events':ti,ab OR 'major adverse cardiac events':ti,ab OR MACE:ti,ab OR 'major adverse limb events':ti,ab OR MALE:ti,ab OR 'antithrombotic therapy':ti,ab OR antithrombotic*:ti,ab OR anticoagul*:ti,ab OR antiplatelet*:ti,ab OR rivaroxaban:ti,ab OR aspirin:ti,ab OR 'dual pathway inhibition':ti,ab OR 'PCSK9 inhibitor*':ti,ab OR evolocumab:ti,ab OR alirocumab:ti,ab OR 'bempedoic acid':ti,ab OR 'GLP-1 receptor agonist*':ti,ab OR semaglutide:ti,ab OR liraglutide:ti,ab OR dulaglutide:ti,ab OR 'SGLT2 inhibitor*':ti,ab OR empagliflozin:ti,ab OR dapagliflozin:ti,ab OR canagliflozin:ti,ab OR 'exercise therapy':ti,ab OR 'supervised exercise':ti,ab OR 'supervised exercise therapy':ti,ab OR SET:ti,ab OR rehabilitation:ti,ab OR 'risk stratification':ti,ab OR 'residual risk':ti,ab OR prognosis:ti,ab OR prognostic:ti,ab OR prediction:ti,ab OR predictor*:ti,ab)
Scopus	TITLE-ABS-KEY("peripheral artery disease" OR "peripheral arterial disease" OR PAD) AND TITLE-ABS-KEY("systemic atherosclerosis" OR "polyvascular disease" OR "polyvascular atherosclerotic disease" OR "multivessel atherosclerosis" OR "major adverse cardiovascular events" OR "major adverse cardiac events" OR MACE OR "major adverse limb events" OR MALE OR "antithrombotic therapy" OR antithrombotic* OR anticoagul* OR antiplatelet* OR rivaroxaban OR aspirin OR "dual pathway inhibition" OR "PCSK9 inhibitor*" OR evolocumab OR alirocumab OR "bempedoic acid" OR "GLP-1 receptor agonist*" OR semaglutide OR liraglutide OR dulaglutide OR "SGLT2 inhibitor*" OR empagliflozin OR dapagliflozin OR canagliflozin OR "exercise therapy" OR "supervised exercise" OR "supervised exercise

Database	Search strategy
	therapy" OR SET OR rehabilitation OR "risk stratification" OR "residual risk" OR prognosis OR prognostic OR prediction OR predictor*)
Web of Science Core Collection	TS=("peripheral artery disease" OR "peripheral arterial disease" OR PAD) AND TS=("systemic atherosclerosis" OR "polyvascular disease" OR "polyvascular atherosclerotic disease" OR "multivessel atherosclerosis" OR "major adverse cardiovascular events" OR "major adverse cardiac events" OR MACE OR "major adverse limb events" OR MALE OR "antithrombotic therapy" OR antithrombotic* OR anticoagul* OR antiplatelet* OR rivaroxaban OR aspirin OR "dual pathway inhibition" OR "PCSK9 inhibitor*" OR evolocumab OR alirocumab OR "bempedoic acid" OR "GLP-1 receptor agonist*" OR semaglutide OR liraglutide OR dulaglutide OR "SGLT2 inhibitor*" OR empagliflozin OR dapagliflozin OR canagliflozin OR "exercise therapy" OR "supervised exercise" OR "supervised exercise therapy" OR SET OR rehabilitation OR "risk stratification" OR "residual risk" OR prognosis OR prognostic OR prediction OR predictor*)

Supplementary Table S1. Database-specific search strategies used for the literature review

Abbreviations: GLP-1, glucagon-like peptide-1; MACE, major adverse cardiovascular events; MALE, major adverse limb events; PAD, peripheral artery disease; PCSK9, proprotein convertase subtilisin/kexin type 9; SET, supervised exercise therapy; SGLT2, sodium-glucose cotransporter-2.

The search covered publications from January 1, 2000, through August 20, 2026. The same conceptual search strategy was adapted to the syntax and field structure of each database. Searches were restricted to title and abstract fields in PubMed/MEDLINE and Embase, whereas equivalent title/abstract/keyword or topic-field searches were performed in Scopus and Web of Science, respectively. The electronic search was supplemented by manual screening of the reference lists of relevant reviews, original studies, guidelines, consensus documents, and landmark publications.
