# Supplementary material for: PAD as a Marker of Systemic Atherosclerosis: A Comprehensive Review of Residual Risk and Contemporary Strategies for MACE and MALE Reduction
Source: Medicina (Kaunas). 2026 Oct 2;62(10):1905. doi: 10.3390/medicina62101905 (PMC13707036; doi:10.3390/medicina62101905)
Supplement: Supplementary file 1 [file medicina-62-01905-s001.zip › Supplementary Table S2.pdf]

Search domain	Terms derived from the global strategy
1. PAD as a marker of systemic and polyvascular atherosclerosis	"systemic atherosclerosis" OR "polyvascular disease" OR "polyvascular atherosclerotic disease" OR "multivessel atherosclerosis"
2. Residual risk, MACE, MALE and prognostic stratification	"major adverse cardiovascular events" OR "major adverse cardiac events" OR MACE OR "major adverse limb events" OR MALE OR "risk stratification" OR "residual risk" OR prognosis OR prognostic OR prediction OR predictor*
3. Contemporary pharmacological risk-reduction strategies	"antithrombotic therapy" OR antithrombotic* OR anticoagul* OR antiplatelet* OR rivaroxaban OR aspirinOR "dual pathway inhibition" OR "PCSK9 inhibitor*" OR evolocumab OR alirocumab OR "bempedoic acid"OR "GLP-1 receptor agonist*" OR semaglutide OR liraglutide OR dulaglutide OR "SGLT2 inhibitor*" OR empagliflozin OR dapagliflozin OR canagliflozin
4. Exercise-based and functional risk-modifying strategies	"exercise therapy" OR "supervised exercise" OR "supervised exercise therapy" OR SET OR rehabilitation
**Each block would always be combined with:**<br><br>("peripheral artery disease" OR "peripheral arterial disease" OR PAD)	

Supplementary Table S2. Concept-driven subdivision of the global search strategy
