# Supplementary material for: PAD as a Marker of Systemic Atherosclerosis: A Comprehensive Review of Residual Risk and Contemporary Strategies for MACE and MALE Reduction
Source: Medicina (Kaunas). 2026 Oct 2;62(10):1905. doi: 10.3390/medicina62101905 (PMC13707036; doi:10.3390/medicina62101905)
Supplement: Supplementary file 1 [file medicina-62-01905-s001.zip › Supplementary_Table_S3_PAD_Evidence_Word.pdf]

Study/source	Design	PAD population	Intervention/comparator	Follow-up	Endpoint definition
**COMPASS PAD subgroup (2018)**	Randomized, double-blind, three-arm trial; PAD subgroup analysis	Symptomatic lower-extremity or carotid PAD, or asymptomatic PAD with concomitant CAD; lower-extremity PAD analysis, n = 5,551	Rivaroxaban 2.5 mg twice daily plus aspirin 100 mg once daily vs rivaroxaban 5 mg twice daily vs aspirin 100 mg once daily	Median ≈21 months; trial terminated early for efficacy	MACE: cardiovascular death, MI, or stroke; MALE assessed as a secondary limb outcome
**VOYAGER PAD (2020)**	Randomized, double-blind, placebo-controlled trial	Symptomatic lower-extremity PAD within 10 days after surgical or endovascular revascularization; n = 6,564	Rivaroxaban 2.5 mg twice daily plus aspirin vs placebo plus aspirin; clopidogrel permitted at the investigator's discretion	Median 28 months	Composite of cardiovascular death, MI, ischemic stroke, acute limb ischemia, or major amputation for vascular causes
**CAPRIE PAD subgroup (1996)**	Randomized, double-blind trial; PAD subgroup analysis	Patients at risk of ischemic events, including a symptomatic PAD subgroup	Clopidogrel 75 mg once daily vs aspirin 325 mg once daily	Mean ≈1.9 years	Composite of ischemic stroke, MI, or vascular death
**EUCLID (2017)**	Randomized, double-blind, active-comparator trial	Symptomatic PAD; n = 13,885	Ticagrelor vs clopidogrel monotherapy	Median ≈30 months	MACE: cardiovascular death, MI, or ischemic stroke
**BEST-CLI, cohort 1 (2022)**	Randomized controlled trial	CLTI with infrainguinal disease and an adequate single-segment great saphenous vein; overall n = 1,830; cohort 1, n = 1,434	Surgical bypass vs endovascular therapy	Median 2.7 years	Primary endpoint: MALE (above-ankle amputation or major limb reintervention) or death from any cause
**FOURIER PAD subgroup (2018)**	Randomized, placebo-controlled trial; prespecified PAD subgroup analysis	Patients with established ASCVD, including a PAD subgroup	Evolocumab plus statin therapy vs placebo plus statin therapy	Median ≈2.2 years	MACE; MALE evaluated as an exploratory outcome
**ODYSSEY OUTCOMES PAD analysis (2018)**	Randomized, placebo-controlled trial; PAD subgroup/secondary analysis	Patients with recent acute coronary syndrome and known PAD	Alirocumab plus intensive or maximally tolerated statin therapy vs placebo plus statin therapy	Median 2.8 years	MACE; PAD events included progression to CLTI, limb revascularization, or unplanned amputation for ischemia
**Heart Protection Study PAD subgroup (2002)**	Randomized, placebo-controlled trial; PAD subgroup analysis	Patients with PAD, with or without concomitant CAD	Simvastatin 40 mg once daily vs placebo	Mean ≈5 years	Major vascular events: coronary death, MI, stroke, or revascularization

Abbreviations: ACS, acute coronary syndrome; ALI, acute limb ischemia; ASCVD, atherosclerotic cardiovascular disease; CAD, coronary artery disease; CI, confidence interval; CLTI, chronic limb-threatening ischemia; CV, cardiovascular; HR, hazard ratio; ISTH, International Society on Thrombosis and Haemostasis; MACE, major adverse cardiovascular events; MALE, major adverse limb events; MI, myocardial infarction; PAD, peripheral artery disease; TIMI, Thrombolysis in Myocardial Infarction.

Supplementary Table S3. Principal clinical evidence informing therapeutic strategies in peripheral artery disease

Structured narrative synthesis of major trials and clinically relevant subgroup analyses

Panel A. Study design, population, intervention, and endpoint definitions

Study/source	MACE	MALE	Mortality	Major bleeding	Functional outcomes	Key limitations and interpretive confidence
**COMPASS PAD subgroup (2018)**	Rivaroxaban plus aspirin vs aspirin: HR 0.72 (p = 0.005); PAD subgroup: HR 0.70 (95% CI 0.56–0.88; p = 0.002)	Lower incidence with rivaroxaban plus aspirin vs aspirin alone (≈1% vs ≈2%; p = 0.004)	Lower all-cause mortality in the overall trial population	Major bleeding increased (≈3% vs ≈2%); no significant excess of intracranial or fatal bleeding	Not assessed as a primary endpoint	Subgroup analysis not powered specifically for PAD outcomes; early termination may have increased the estimated treatment effect; selected patients with prior stroke or high bleeding risk were excluded. Moderate-to-high interpretive confidence.
**VOYAGER PAD (2020)**	Primary composite: 17.3% vs 19.9%; HR 0.85 (95% CI 0.76–0.96; p = 0.009)	Reduced, driven principally by fewer acute limb ischemic events; major amputation alone was not significantly reduced	No significant difference in all-cause mortality	TIMI major bleeding: 2.65% vs 1.87%; HR 1.43 (95% CI 0.97–2.10; p = 0.07). ISTH major bleeding was significantly increased; no excess of intracranial or fatal bleeding	Not assessed as a primary endpoint	Dedicated post-revascularization PAD population; findings are not directly generalizable to stable PAD without recent revascularization. High interpretive confidence for the primary composite outcome.
**CAPRIE PAD subgroup (1996)**	Annual event rate 3.71% vs 4.86%; relative risk reduction 23.8% (p = 0.0028) in the PAD subgroup	Not assessed as a dedicated endpoint	No significant mortality difference demonstrated	Overall safety was broadly similar between treatment groups	Not assessed	Subgroup finding; the trial was not powered specifically for PAD and the subgroup result should be interpreted cautiously. Moderate interpretive confidence.
**EUCLID (2017)**	HR 1.02 (95% CI 0.92–1.13); ticagrelor was not superior to clopidogrel	No significant between-group difference	No significant between-group difference	Major bleeding rates were similar between groups	Not a primary endpoint	Methodologically robust dedicated PAD trial with a neutral primary result. High interpretive confidence for absence of ticagrelor superiority.
**BEST-CLI, cohort 1 (2022)**	Similar at 30 days and during follow-up; follow-up HR 0.94, not statistically significant	Primary composite event: 42.6% vs 57.4%; HR 0.68 (95% CI 0.59–0.79; p < 0.001), driven mainly by fewer major reinterventions	No significant difference in all-cause mortality (HR 0.98)	Not a principal study endpoint	Ambulation and quality of life were not primary components of the composite endpoint	The benefit applies to patients with an adequate great saphenous vein conduit and should not be extrapolated to patients without suitable autologous vein. High interpretive confidence within the cohort 1 population.
**FOURIER PAD subgroup (2018)**	HR 0.79 (95% CI 0.66–0.94; p = 0.0098) in the PAD subgroup	HR 0.63 (95% CI 0.39–1.03; p = 0.063) in the PAD subgroup; nonsignificant trend	No PAD-specific mortality effect established	No significant increase in major bleeding	Not assessed	Prespecified subgroup analysis; the PAD-specific MALE estimate did not reach statistical significance. Moderate interpretive confidence.
**ODYSSEY OUTCOMES PAD analysis (2018)**	MACE was reduced in the overall trial population	PAD events: HR 0.59 (95% CI 0.40–0.86)	All-cause mortality was reduced in the overall trial population	No significant increase in major bleeding	Not assessed	Post-acute coronary syndrome population; PAD-specific limb findings derive from a secondary analysis and require cautious interpretation. Moderate interpretive confidence.
**Heart Protection Study PAD subgroup (2002)**	Approximately 24% proportional reduction in major vascular events in participants with PAD	Peripheral vascular events were reduced	Vascular mortality was reduced in the overall trial population	No clinically relevant excess of major adverse safety events	Not assessed	Large and robust subgroup supporting statin therapy in PAD, although the analysis was not a dedicated PAD trial. Moderate-to-high interpretive confidence.

Interpretive confidence represents a qualitative narrative judgment based on study design, directness to the PAD population, endpoint relevance, precision, and applicability; it does not constitute a formal GRADE assessment.

Supplementary Table S3. Principal clinical evidence informing therapeutic strategies in peripheral artery disease continued

Panel B. Clinical outcomes and interpretive limitations
