# Supplementary material for: PAD as a Marker of Systemic Atherosclerosis: A Comprehensive Review of Residual Risk and Contemporary Strategies for MACE and MALE Reduction
Source: Medicina (Kaunas). 2026 Oct 2;62(10):1905. doi: 10.3390/medicina62101905 (PMC13707036; doi:10.3390/medicina62101905)
Supplement: Supplementary file 1 [file medicina-62-01905-s001.zip › Supplementary_Table_S4_Metabolic_Exercise_PAD_Word.pdf]

Study/source	Design	PAD population	Intervention/comparator	Follow-up	Endpoint definition
**STRIDE (2025)**	Phase 3, randomized, double-blind, placebo-controlled trial; first GLP-1 receptor agonist trial conducted in a primary PAD population	Symptomatic PAD and type 2 diabetes mellitus with intermittent claudication (Fontaine stage IIa); n = 792	Once-weekly subcutaneous semaglutide vs placebo	52 weeks	Primary endpoint: ratio to baseline in maximum walking distance; cardiovascular and limb events were exploratory
**SOUL (2025)**	Randomized, double-blind, placebo-controlled cardiovascular outcomes trial; PAD subgroup and limb-outcome analyses	Type 2 diabetes mellitus with established atherosclerotic cardiovascular disease and/or chronic kidney disease; PAD subgroup	Oral semaglutide vs placebo in addition to standard care	Multiyear follow-up	Primary endpoint: MACE; hospitalization for acute or chronic limb ischemia evaluated in a secondary/prespecified analysis
**SUSTAIN-6 PAD subgroup (2016)**	Randomized, double-blind, placebo-controlled cardiovascular outcomes trial; prespecified subgroup analysis	Type 2 diabetes mellitus at high cardiovascular risk; subgroup with PAD	Subcutaneous semaglutide vs placebo	Approximately 2.1 years	MACE: cardiovascular death, nonfatal myocardial infarction, or nonfatal stroke
**LEADER PAD subgroup (2016)**	Randomized, double-blind, placebo-controlled cardiovascular outcomes trial; prespecified PAD subgroup with post hoc amputation analysis	Type 2 diabetes mellitus at high cardiovascular risk; subgroup with PAD	Liraglutide vs placebo	Approximately 3.8 years	MACE; lower-extremity amputation assessed post hoc
**EMPA-REG OUTCOME PAD subgroup (2015)**	Randomized, double-blind, placebo-controlled cardiovascular outcomes trial; PAD subgroup analysis	Type 2 diabetes mellitus and established cardiovascular disease; PAD subgroup, n ≈ 1,462 (20.8%)	Empagliflozin vs placebo	Approximately 3.1 years	MACE and cardiovascular mortality; limb outcomes evaluated in the PAD analysis
**CANVAS Program PAD subgroup (2017)**	Randomized, double-blind, placebo-controlled cardiovascular outcomes program; PAD subgroup analysis	Type 2 diabetes mellitus at high cardiovascular risk; approximately 21% had PAD	Canagliflozin vs placebo	Approximately 3.6 years	MACE and lower-limb amputation
**DECLARE–TIMI 58 (2019)**	Randomized, double-blind, placebo-controlled cardiovascular outcomes trial; PAD subgroup/secondary analyses	Type 2 diabetes mellitus with established cardiovascular disease or multiple risk factors; PAD subgroup	Dapagliflozin vs placebo	Approximately 4.2 years	Co-primary endpoints: MACE and cardiovascular death or hospitalization for heart failure; limb outcomes assessed separately
**SGLT2 inhibitor meta-analysis in PAD (Hu et al., 2026)**	Systematic review and meta-analysis of randomized cardiovascular outcomes trials; five studies and 7,275 participants with PAD	Participants with PAD enrolled in SGLT2 inhibitor cardiovascular outcomes trials	SGLT2 inhibitor vs placebo	Variable across included trials	MACE, cardiovascular death or hospitalization for heart failure, all-cause mortality, and lower-limb amputation

Supplementary Table S4. Clinical evidence supporting metabolic and exercise-based strategies in peripheral artery disease

Structured summary distinguishing dedicated PAD trials from subgroup, secondary, and exploratory evidence

Panel A1. Metabolic strategies: study design, population, intervention, and endpoint definitions

Study/source	MACE	MALE	Mortality	Major bleeding	Functional outcomes	Key limitations and interpretive confidence
**STRIDE (2025)**	The trial was not powered to establish an effect on MACE	The trial was not powered to establish an effect on MALE	Mortality was not an efficacy endpoint; no treatment-related deaths were reported	Not reported as a dedicated efficacy or safety endpoint	Maximum walking distance: estimated treatment ratio 1.13 (95% CI 1.06–1.21; p = 0.0004), corresponding to an estimated between-group difference of 39.9 m; pain-free walking distance, quality of life, and ankle–brachial index also improved	Dedicated PAD trial with a clinically relevant functional primary endpoint; hard cardiovascular and limb outcomes were exploratory and should not be used to infer event reduction. High confidence for functional efficacy; low confidence for MACE/MALE.
**SOUL (2025)**	Oral semaglutide reduced MACE in the overall trial population; PAD-specific estimates should be interpreted as subgroup evidence	Hospitalization for acute or chronic limb ischemia was lower in the relevant analysis (approximately 30% relative reduction)	No PAD-specific mortality effect was established	Not reported as a PAD-specific outcome	Not assessed as a primary endpoint	Large cardiovascular outcomes trial, but the limb finding was not the primary trial endpoint and the PAD analysis does not provide the same level of directness as a dedicated PAD trial. Moderate interpretive confidence.
**SUSTAIN-6 PAD subgroup (2016)**	PAD subgroup: HR 0.61 (95% CI 0.33–1.13); absolute risk reduction 4.6%; the estimate was not statistically significant	No direct PAD-specific effect on MALE was established	No PAD-specific mortality effect was established	Not reported as a PAD-specific outcome	Not assessed	Subgroup analysis with limited statistical power; relative effects were consistent across PAD strata (interaction p = 0.49). Moderate interpretive confidence for consistency, but low confidence for a PAD-specific treatment effect.
**LEADER PAD subgroup (2016)**	PAD subgroup: HR 0.77 (95% CI 0.58–1.01); absolute risk reduction 4.1%; the estimate was not statistically significant	Lower-extremity amputations: HR 0.65 (95% CI 0.45–0.95) in a post hoc analysis	No PAD-specific mortality effect was established	Not reported as a PAD-specific outcome	Not assessed	MACE derives from a subgroup analysis and the amputation finding from a post hoc analysis; neither establishes a class-wide limb benefit. Moderate interpretive confidence.
**EMPA-REG OUTCOME PAD subgroup (2015)**	MACE was reduced in the overall trial population (10.5% vs 12.1%; p = 0.04); PAD-specific evidence derives from subgroup analysis	No excess of lower-limb amputations was observed; a definitive reduction in MALE was not established	Cardiovascular and all-cause mortality were lower in the overall trial; subgroup findings were directionally consistent	Not a prespecified class-specific safety endpoint	Not assessed	PAD subgroup analysis; the study does not establish a class effect on limb events. Moderate interpretive confidence for cardiovascular outcomes; limited direct evidence for MALE.
**CANVAS Program PAD subgroup (2017)**	Overall trial: 26.9 vs 31.5 events per 1,000 patient-years; HR 0.86 (95% CI 0.75–0.97); effect was consistent by PAD status	No MALE reduction was demonstrated; amputations occurred at 6.3 vs 3.4 per 1,000 patient-years	Cardiovascular benefit was demonstrated in the overall program; no PAD-specific mortality conclusion was established	Not a prespecified class-specific safety endpoint	Not assessed	The amputation signal, predominantly involving toes or metatarsals, was not consistently reproduced in later canagliflozin or other SGLT2 inhibitor trials. Moderate interpretive confidence; drug-specific safety findings should not be generalized uncritically to the class.
**DECLARE–TIMI 58 (2019)**	No significant reduction in MACE	No definitive reduction in MALE and no excess of lower-limb amputation	Cardiovascular death or hospitalization for heart failure was reduced (4.9% vs 5.8%; p = 0.005), principally through fewer heart-failure hospitalizations; no PAD-specific mortality effect was established	Not a prespecified class-specific safety endpoint	Not assessed	Methodologically robust cardiovascular outcomes trial, but PAD-specific and limb findings are secondary; cardiovascular benefit is principally related to heart-failure outcomes rather than MACE. Moderate interpretive confidence for PAD-specific conclusions.
**SGLT2 inhibitor meta-analysis in PAD (Hu et al., 2026)**	HR 0.89 (95% CI 0.75–1.06); not statistically significant	Lower-limb amputation: HR 1.17 (95% CI 0.87–1.56); no statistically significant difference	All-cause mortality: HR 0.86 (95% CI 0.69–1.08); not statistically significant	Not evaluated as a pooled outcome	Not assessed	PAD evidence was derived largely from subgroups of cardiovascular outcomes trials, with heterogeneity in populations and limb-endpoint definitions. The most consistent benefits concerned heart-failure and kidney outcomes rather than MACE or MALE. Moderate interpretive confidence.

Panel A2. Metabolic strategies: clinical outcomes and interpretive limitations

Study/source	Design	PAD population	Intervention/comparator	Follow-up	Endpoint definition
**CLEVER (2012)**	Three-arm randomized controlled trial	Symptomatic aortoiliac PAD with intermittent claudication; n = 111	Optimal medical care alone vs stent revascularization plus optimal medical care vs supervised exercise therapy plus optimal medical care	6–18 months	Primary functional endpoint: peak walking time
**ERASE (2015)**	Randomized controlled trial	PAD with intermittent claudication	Endovascular revascularization plus supervised exercise therapy vs supervised exercise therapy alone	12 months	Primary functional endpoints: maximum and pain-free walking distance
**LITE (2021)**	Randomized controlled trial	Symptomatic PAD; n = 305	Usual care vs structured home-based high-intensity walking vs structured home-based low-intensity walking	12 months	Primary functional endpoint: change in 6-minute walk distance
**Individual-participant-data meta-analysis of home-based exercise (Thangada et al., 2023)**	Individual-participant-data meta-analysis of structured exercise trials, including GOALS and LITE	Symptomatic PAD and PAD with atypical lower-extremity symptoms	Structured home-based walking exercise vs nonexercise control	6–12 months	Functional endpoints: 6-minute walk distance and Walking Impairment Questionnaire domains

Panel B1. Exercise-based strategies: study design, population, intervention, and endpoint definitions

Study/source	MACE	MALE	Mortality	Major bleeding	Functional outcomes	Key limitations and interpretive confidence
**CLEVER (2012)**	Not assessed	Not assessed	Not assessed	Not applicable	Supervised exercise produced the greatest improvement in peak walking time; disease-specific quality-of-life improvement was greater with stent revascularization	Small sample and no hard cardiovascular or limb-event endpoints; direct, randomized evidence for functional outcomes. High interpretive confidence for walking performance, but no inference can be made regarding MACE or MALE.
**ERASE (2015)**	Not assessed	Not assessed	Not assessed	Not applicable	Combination therapy produced greater gains in maximum walking distance (between-group difference, 282 m) and pain-free walking distance (408 m) than supervised exercise alone	Focused on functional outcomes and not powered for MACE, MALE, or mortality. High interpretive confidence for short-term walking outcomes.
**LITE (2021)**	Not assessed	Not assessed	Not assessed	Not applicable	High-intensity walking improved 6-minute walk distance; low-intensity walking did not improve the primary outcome compared with usual care	Direct randomized evidence for walking performance; no cardiovascular or limb-event endpoints. High interpretive confidence for the intensity-dependent functional effect.
**Individual-participant-data meta-analysis of home-based exercise (Thangada et al., 2023)**	Not assessed	Not assessed	Not assessed	Not applicable	Structured home-based exercise improved 6-minute walk distance by approximately 56 m compared with control and improved Walking Impairment Questionnaire domains	Evidence is limited to functional outcomes; no reduction in MACE or MALE was established. Moderate-to-high interpretive confidence for functional benefit, subject to between-trial heterogeneity.

Interpretive confidence is a qualitative narrative judgment based on study design, directness to the PAD population, endpoint relevance, precision, and applicability; it does not constitute a formal GRADE assessment. Statements concerning subgroup, secondary, exploratory, and post hoc findings have been explicitly identified and should not be interpreted as definitive PAD-specific treatment effects.

Abbreviations: ABI, ankle–brachial index; CI, confidence interval; CV, cardiovascular; CVOT, cardiovascular outcomes trial; GLP-1, glucagon-like peptide-1; HF, heart failure; HR, hazard ratio; MACE, major adverse cardiovascular events; MALE, major adverse limb events; MI, myocardial infarction; PAD, peripheral artery disease; QoL, quality of life; SGLT2, sodium–glucose cotransporter 2; T2DM, type 2 diabetes mellitus; WIQ, Walking Impairment Questionnaire; 6MWD, 6-minute walk distance.

Panel B2. Exercise-based strategies: clinical outcomes and interpretive limitations
